# Supplementary material for: CDK4/6 inhibition triggers ICAM1-driven immune response and sensitizes LKB1 mutant lung cancer to immunotherapy
Source: Nat Commun. 2023 Mar 4;14:1247. doi: 10.1038/s41467-023-36892-4 (PMC9985635; doi:10.1038/s41467-023-36892-4)
Supplement: Supplementary file 1 — Supplementary Information [file 41467_2023_36892_MOESM1_ESM.pdf]

CDK4/6 inhibition triggers ICAM1-driven immune response and sensitizes *LKB1* mutant lung cancer to immunotherapy

SUPPLEMENTAL FIGURES

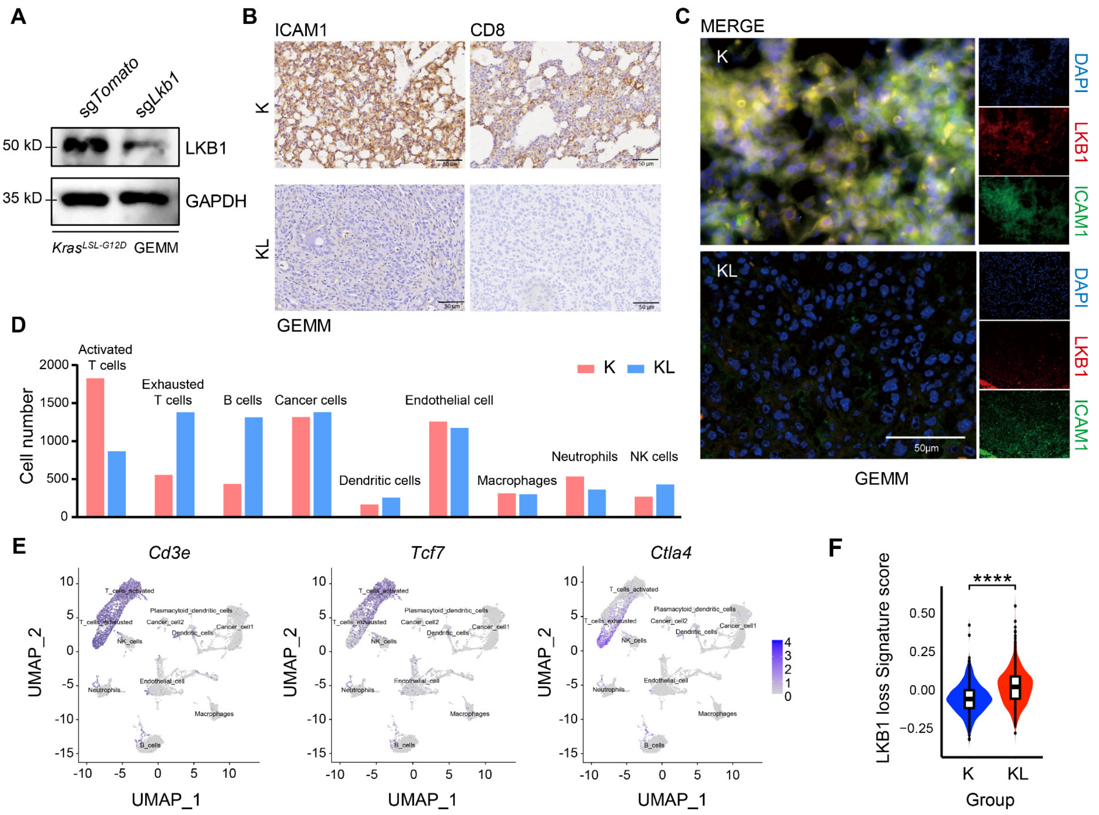

Supplementary Figure 1 (Related with Figure 1) *LKB1* deficiency in lung cancer dictates the TME. (A) Immunoblot analysis of lysates from lung tumor tissues of *Kras*$^{G12D/+}$ mice virally infected with pSECC-*sgLkb1* (KL) or pSECC-*sgTomato* (K). n = 3 independent experiments. (B) Representative images of IHC staining of K and KL lung tumors using anti-CD8α and anti-ICAM1 antibodies. Scale bar, 50 μm (40×). n = 5 biologically independent mice examined over 1 independent experiment. (C) Representative immunofluorescence images of K and KL lung tumors using anti-LKB1 and anti-ICAM1 antibodies. Scale bar, 50 μm (40×). n = 5 biologically independent mice examined over 1 independent experiment. (D) Absolute numbers of cells from K and KL samples for clusters identified in **Figure 1.** (E) UMAP plot for the expression of *Cd3e*, *Tcf7* and *Ctla4* in the scRNA-seq datasets. (F) Gene set variation analysis with *LKB1* loss gene set for cells in cancer_cells1 and cancer_cells2 cluster in K (n = 662 cancer cells) and KL (n = 701 cancer cells) samples. ****p < 0.0001.

(Results are presented as violin plot (median, 25%-75%, range). Two-tailed Student's t-test was used to compare the variables of two groups. ****p<.0001. Source data are provided as a Source Data file.)

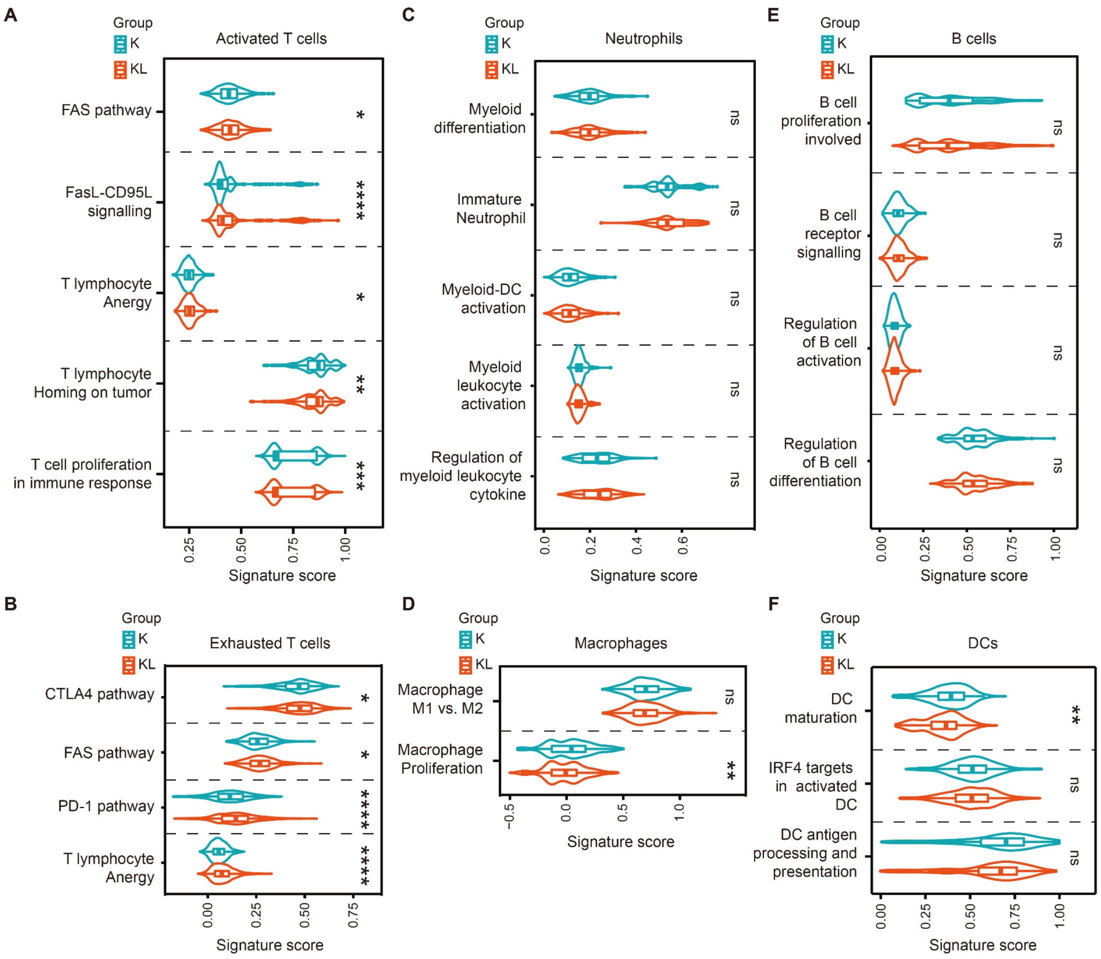

Supplementary Figure 2 (**Related with Figure 1**) Gene set variation analysis with immune-related signatures for cells in the (A) activated T cells cluster (n = 1829 cells for K, n = 867 cells for KL), *p = 0.0306, ****p < 0.0001, *p = 0.0104, **p = 0.0029, ***p = 0.0002. (B) exhausted T cells cluster (n = 557 cells for K, n = 1380 cells for KL), *p = 0.0299, *p = 0.0196, ****p < 0.0001, ****p < 0.0001. (C) neutrophils cluster (n = 535 cells for K, n = 366 cells for KL). (D) macrophages cluster (n = 314 cells for K, n = 302 cells for KL), **p = 0.0055. (E) B cells cluster (n = 437 cells for K, n = 1316 cells for KL) and (F) DCs cluster (n = 168 cells for K, n = 259 cells for KL), **p = 0.0012.

(Data are presented as violin plot (median, 25%-75%, range). Statistical significance was tested with a two-tailed Mann–Whitney U test. n.s., not significant; *p<.05; **p<.01; ****p<.0001. Source data are provided as a Source Data file.)

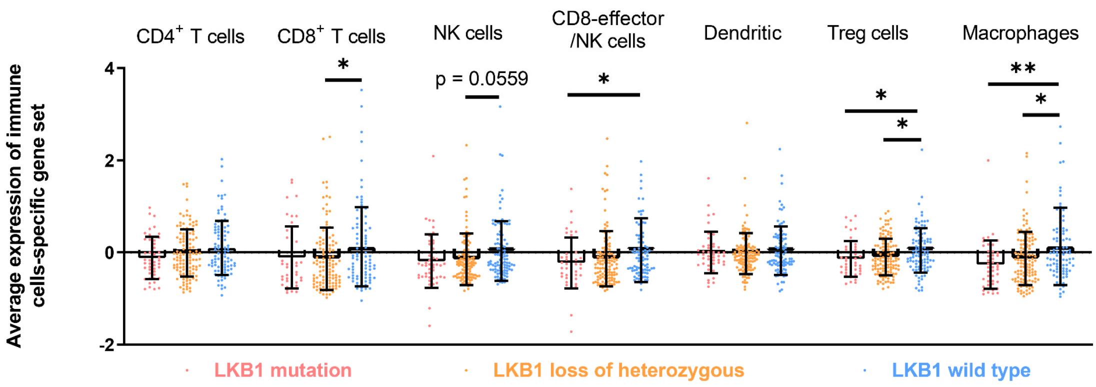

Supplementary Figure 3 (**Related with Figure 1**) The immune profiles of tumors under different *LKB1* statuses. Average expression of immune cell-specific gene sets across the TCGA-LUAD patient cohort harboring different statuses of *LKB1*. n = 45 of patients harboring *LKB1* mutation, n = 120 of patients with *LKB1* loss of heterozygous, n = 97 in *LKB1* wild type patients. *p = 0.0323, *p = 0.0431, *p = 0.0396, *p = 0.0303, **p = 0.0042, *p = 0.0141. (Results are presented as mean ± SEM. One-way ANOVA followed by Tukey's multiple comparisons test was performed for multi-group comparisons. n.s., not significant; *p < .05; **p < .01. Source data are provided as a Source Data file.)

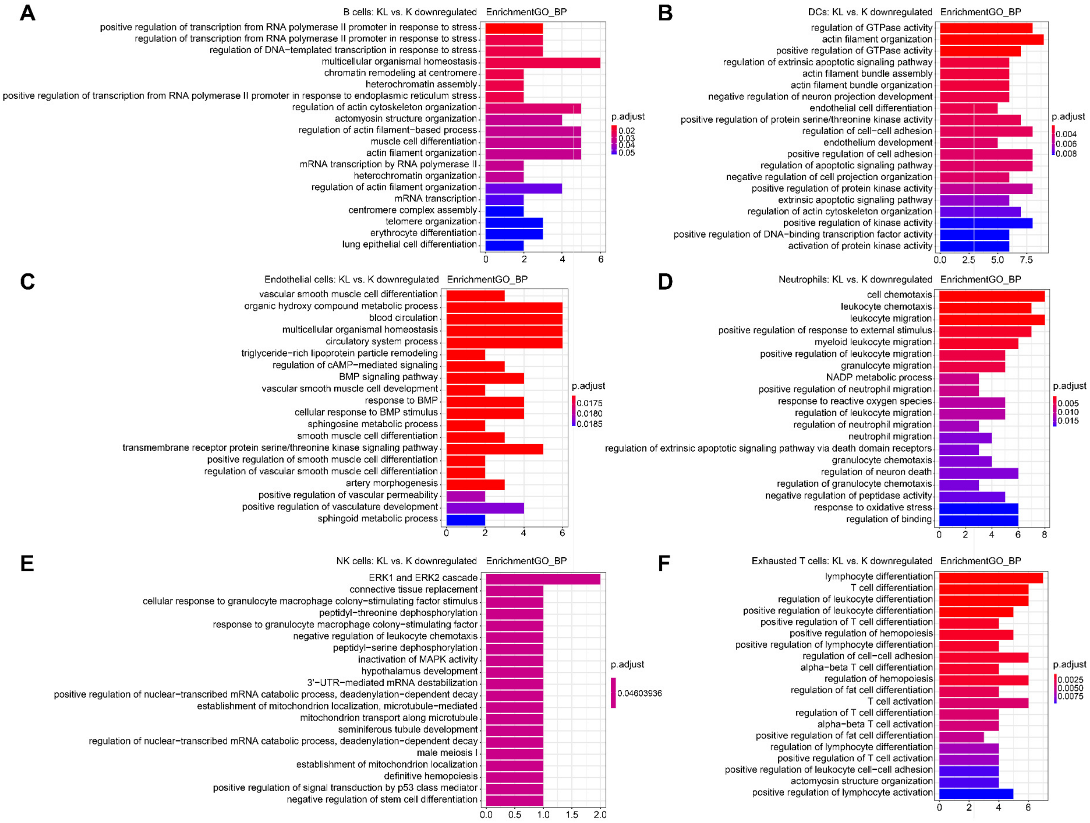

Supplementary Figure 4 (Related with Figure 2) Enrichment analysis on DEGs between KL versus K in (A) B cells cluster, (B) Dendritic cells cluster, (C) Endothelial cells cluster, (D) Neutrophils cluster, (E) NK cells cluster and (F) Exhausted T cells clusters, using clusterProfiler for gene sets in GO terms. Statistical significance was tested with one-sided hypergeometric test followed by Benjamini–Hochberg (BH) multiple comparisons test. Source data are provided as a Source Data file.

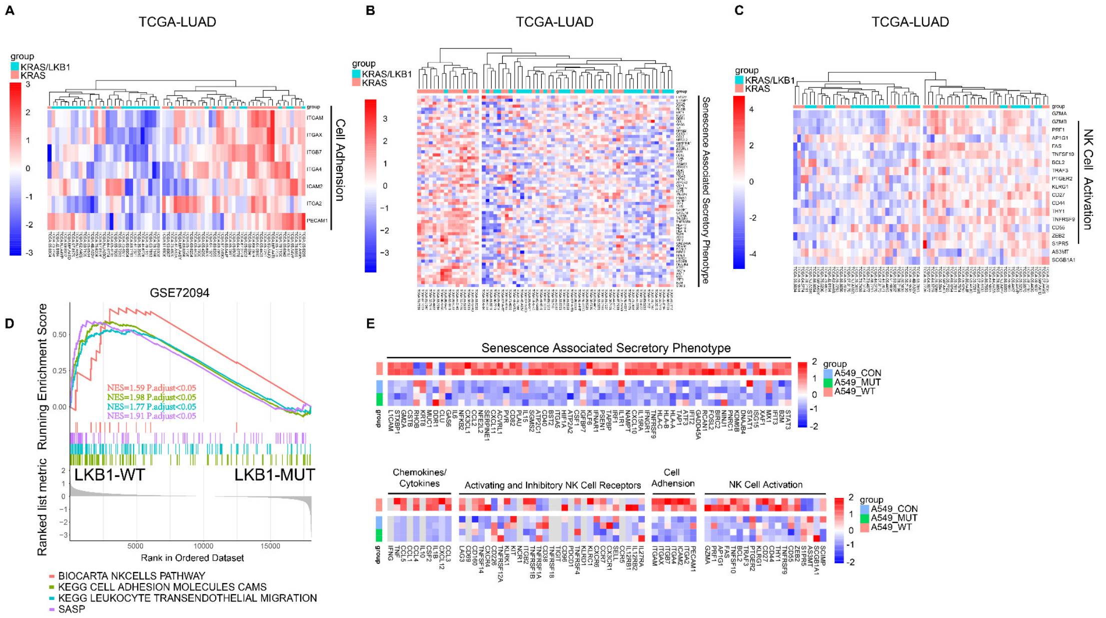

Supplementary Figure 5 (Related with Figure 2) Pathways differently expressed in *KRAS/LKB1* mutation versus *KRAS* mutation groups. (A-C) Heatmap of the indicated pathways differently expressed in TCGA-LUAD patients with *KRAS/LKB1* mutation and *KRAS* mutation. *KRAS/LKB1*, n = 38, *KRAS*, n = 119. (D) Gene-set enrichment analysis (GSEA) of the human lung adenocarcinoma expression profiles (GSE72094) between *LKB1*-MUT (n = 68 samples) and *LKB1*-WT (n = 374 samples) group. Statistical significance was tested with two-sided permutation test, followed by adjustment for multiple hypothesis testing. KEGG CELL ADHESION MOLECULES CAMS, p.adjust = 0.0019; KEGG LEUKOCYTE TRANSENDOTHELIAL MIGRATION, p.adjust = 0.0019; SASP, p.adjust = 0.0019; BIOCARTA NKCELLS PATHWAY, p.adjust = 0.0032. (E) Heatmap of selected pathways differently expressed among *LKB1* intact cells (A549_WT) and *LKB1* deficient cells (A549_CON and A549_MUT). n = 2 samples in each group.

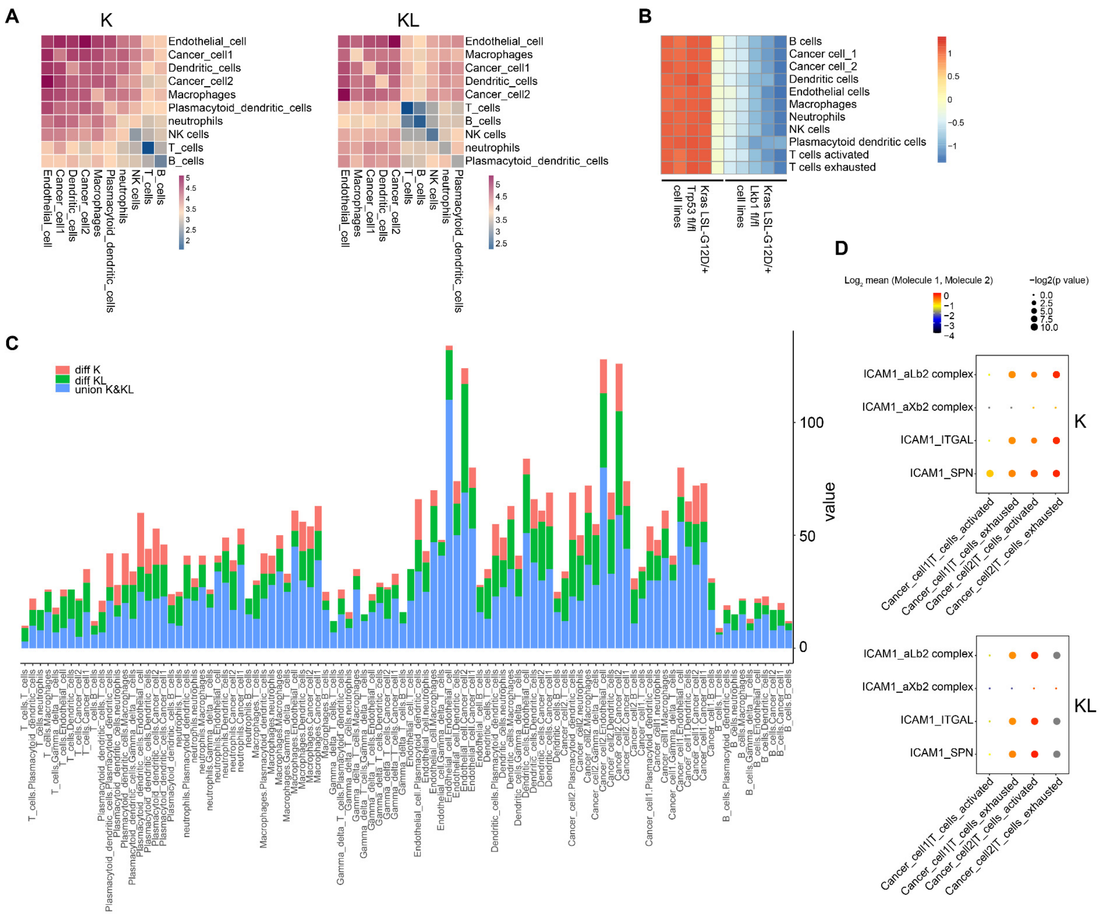

Supplementary Figure 6 (Related with Figure 2) Cell-cell interaction analysis among different cell types in the K versus KL. (A) Heatmap of the CellPhoneDB interactions among different cell types in the TME of K (left) and KL (right). (B) The communication signature scores of *KRAS*^{mutant}/*TP53*^{mutant} (KP, n = 5) and *KRAS*^{mutant}/*LKB1*^{mutant} (KL, n = 5) cancer cells with different cell types (GSE137396). (C) Barplot of per cell-to-cell type interactions, the number of interactions shared and unique in KL versus K tumor samples. (D) Interactions of ICAM1 and ICAM1 receptors between cancer cells and T cells. The statistical test was performed first by randomly permuting the cluster labels of all cells 1000 times, calculating the mean of the average receptor expression level in a cluster and the average ligand expression level in the interacting cluster and generating a null distribution for each receptor-ligand pair in each pairwise comparison between two cell types. Then a p-value to evaluate the probability of cell-type specificity of a given receptor-ligand complex is defined by calculating the proportion of the means which are equal to or higher than the actual mean. Source data are provided as a Source Data file.

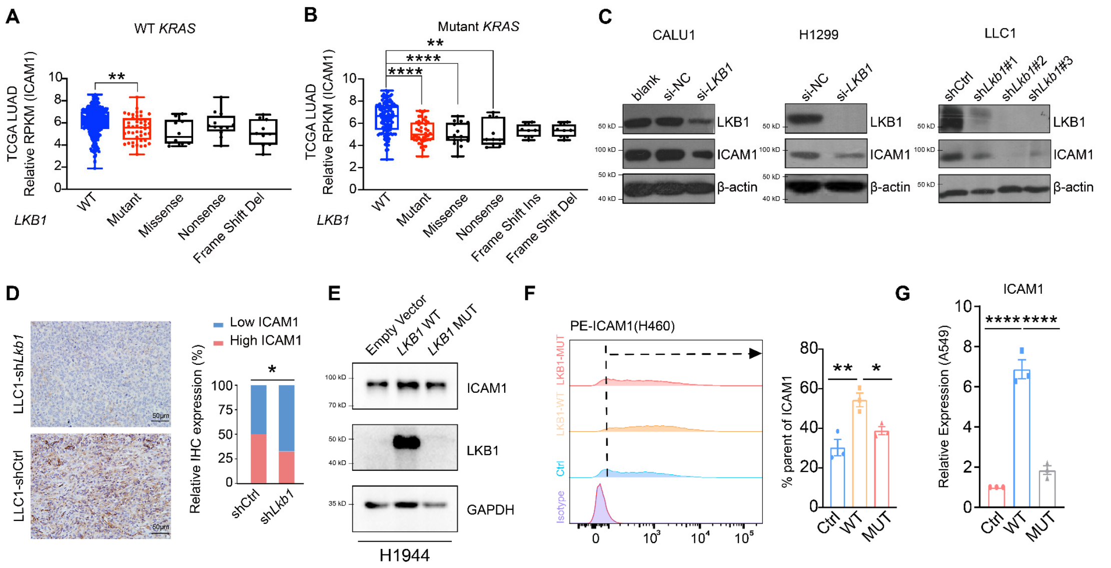

Supplementary Figure 7 (**Related with Figure 2**) LKB1 regulates ICAM1 expression, regardless of the existence of *KRAS* co-mutation. (A-B) Analyses of correlation between *ICAM1* expression and different *LKB1* mutation types, with or without *KRAS* co-mutation using TCGA-LUAD dataset. WT *KRAS* samples (A), n = 321 for WT, n = 44 for Mutant, n = 10 for Missense, n = 12 for Nonsense, n = 10 for Frame Shift Del. **p = 0.0279. Mutant *KRAS* samples (B), n = 120 for WT, n = 44 for Mutant, n = 16 for Missense, n = 10 for Nonsense, n = 8 for Frame Shift Ins, n = 8 for Frame Shift Del. ****p < 0.001, ****p < 0.001, **p = 0.0045. (C) Western blot showing ICAM1 expression of lung cancer cell lines (CALU1, H1299 and LLC1) treated with or without small interference or short hairpin RNA mediated knocking down of *LKB1*/*Lkb1*. n = 3 independent experiments. (D) Representative images and quantification of IHC staining using anti-ICAM1 antibodies for murine LLC1-shCtrl and LLC1-sh*Lkb1* tumors. n = 3 independent experiments. Two-tailed Fisher's exact test was used to analyze the data. *p = 0.0214. Scale bar, 50 µm. (E-G) Analysis of ICAM1 expression of lung cancer cell lines (H1944, H460 and A549) expressing WT (LKB1-WT), mutated (kinase-dead mutation; LKB1-MUT), or no LKB1 (empty vector). Western blot (E), flow cytometry (F), and RT-qPCR (G) analysis of ICAM1 expression. n = 3 independent experiments (E). n = 3 biologically independent samples examined over 1 independent experiment, ** p = 0.0052, *p = 0.0374 (F). n = 3 biologically independent samples examined over 1 independent experiment; ****p < 0.0001, ****p < 0.0001 (G).

(Data are presented as box plot (median, 25%-75%, range) or mean ± SEM. One-way ANOVA followed by Tukey's multiple comparisons test was used to analyze the data unless stated. *p < .05; **p < .01; ***p < .001; ****p < .0001. Source data are provided as a Source Data file.)

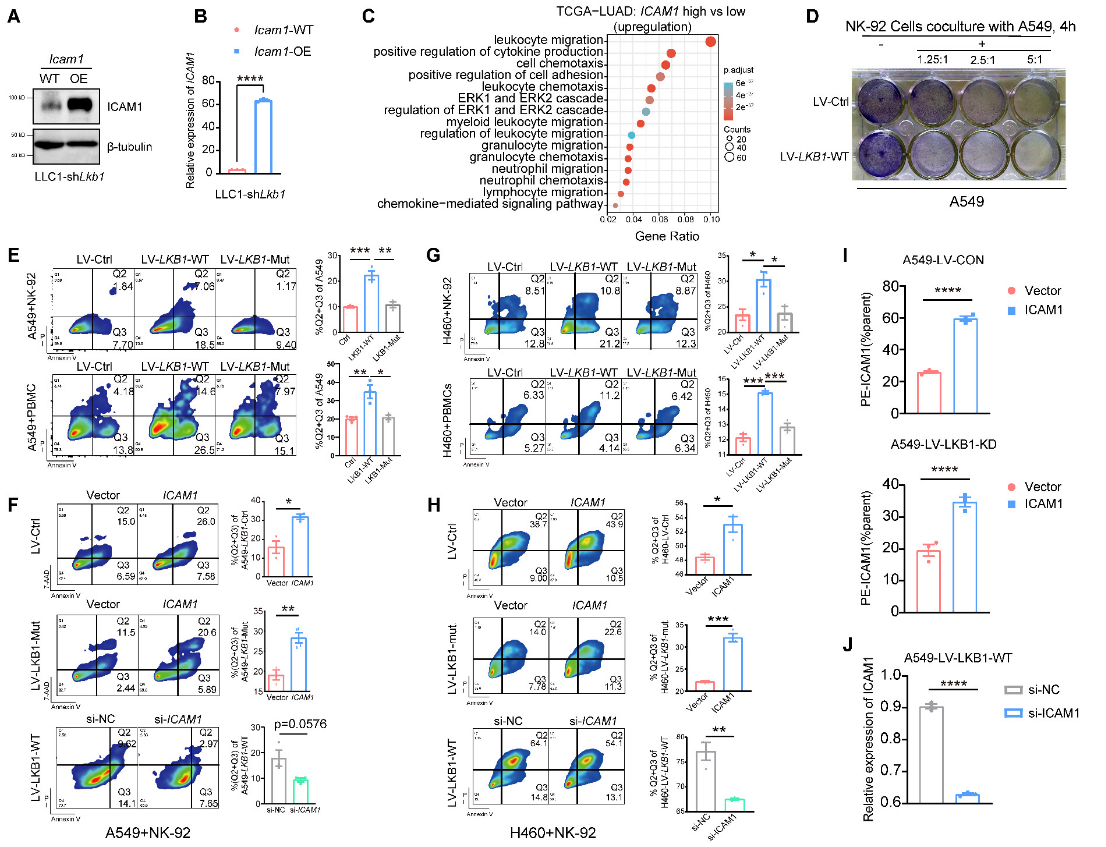

Supplementary Figure 8 (**Related with Figure 3**) ICAM1 functions by modulating cytotoxic immune cell attack of tumor cells. (A-B) Verification of *Icam1* overexpression in Lewis lung cancer cells with *Lkb1* knockdown by western blot (A, n = 3 independent experiment) and RT-qPCR (B, n = 3 samples; ****p < 0.0001). (C) Bubble plot of pathways differently expressed between *ICAM1*-high group (n = 239) and *ICAM1*-low group (n = 239). (D) NK cell-mediated cytotoxicity assay results. A549 cells with or without ectopic *LKB1* expression co-cultured with NK-92 cell for 4h were subjected to crystal violet staining. n = 3 independent experiments. Flow cytometry analysis of apoptosis by Annexin V/PI staining in the A549 (E, n = 3 samples in each group; ***p = 0.0009, **p = 0.0013, **p = 0.0092, *p = 0.0117) and H460 (G, n = 3 samples in each group; *p = 0.0203, *p = 0.0243, ***p = 0.0001, ***p = 0.0006) cell line with different *LKB1* status co-cultured with human NK-92 cells or activated peripheral blood mononuclear cells (PBMCs) from volunteer blood donors for 4h. The E: T ratio (effector to target) was 1.25: 1 for NK-92 cells and 40: 1 for PBMCs. Representative images (left) and quantification results (right) were shown. Assessment of apoptosis in *ICAM1*-overexpressing or *ICAM1*-knockdown A549 cell line (F, samples with LKB1-Mut and LKB1 over-expression, n = 4; others, n = 3; *p = 0.0103, **p = 0.0037) and H460 cell line (H, n = 3 samples in each group; *p = 0.0169, ***p = 0.0004, **p = 0.0058). Cancer cells were co-cultured with human NK-92 cells for 4h. The E: T ratio (effector to target) was 1.25: 1. Representative images (left) and quantification results (right) were shown. (I) Flow cytometry analysis of ICAM1 rescue assay in A549 cells with complete loss of *LKB1* (Ctrl)

or with kinase-dead *LKB1* expression (MUT). n = 3 biologically independent samples examined over 1 independent experiment; ****p < 0.0001, ****p < 0.0001. (J) A549 cells with ectopic wild-type *LKB1* expression were transfected with siRNA targeting *ICAM1* or with scrambled negative control siRNA. Quantitative RT-qPCR analysis of *ICAM1* levels was shown. n = 3 biologically independent samples examined over 1 independent experiment; ****p < 0.0001.

(Results are presented as mean ± SEM. Two-tailed Student's t-test was used to compare the variables of two groups, and one-way ANOVA followed by Tukey's multiple comparisons test was performed for multi-group comparisons. *p < .05; **p < .01; ***p<.001; ****p<.0001. Source data are provided as a Source Data file.)

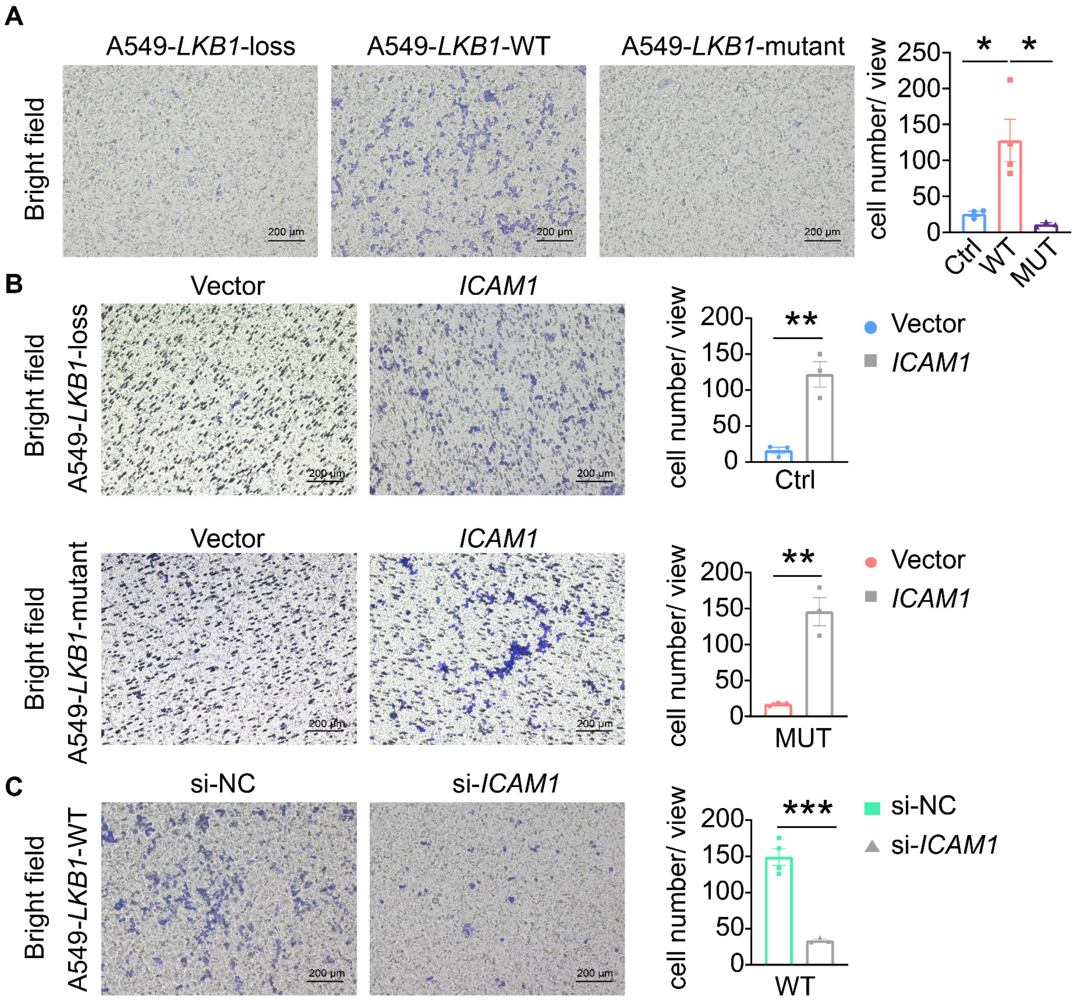

Supplementary Figure 9 (**Related with Figure 3**) T cell migration assay of A549 cell lines. (A) Conditioned medium from A549 parental cells (*LKB1*-loss, n = 3) or *LKB1*-expressing A549 cells (*LKB1*-WT, n = 4; *LKB1*-mutant, n = 3) were collected and filled in the bottom well. Activated PBMCs were loaded into the top chamber of transwell inserts. PBMCs that migrated to the bottom layer were stained by crystal violet blue. Representative images (left) and quantification (right) were shown. *p = 0.0248, *p = 0.0131. Scale bar, 200 μm (20×). (B) Overexpression of *ICAM1* was conducted in *LKB1*-loss and *LKB1*-mutant A549 cells, and (C) knockdown of *ICAM1* was performed in *LKB1*-WT A549 cells. These cells were subsequently subjected to T cell migration assay. Representative images (left) and quantification (right) were shown. n = 3 biologically independent samples examined over 1 independent experiment; **p = 0.0045, **p = 0.0027 (B). n = 4 samples in si-NC and n = 3 samples in si-*ICAM1* groups examined over 1 independent experiment; ***p = 0.0004 (C). Scale bar, 200 μm (20×).

(Results are presented as mean ± SEM. Two-tailed Student's t-test was used to compare the variables of two groups, and one-way ANOVA followed by Tukey's multiple comparisons test was performed for multi-group comparisons. *p < .05; **p < .01; ***p < .001. Source data are provided as a Source Data file.)

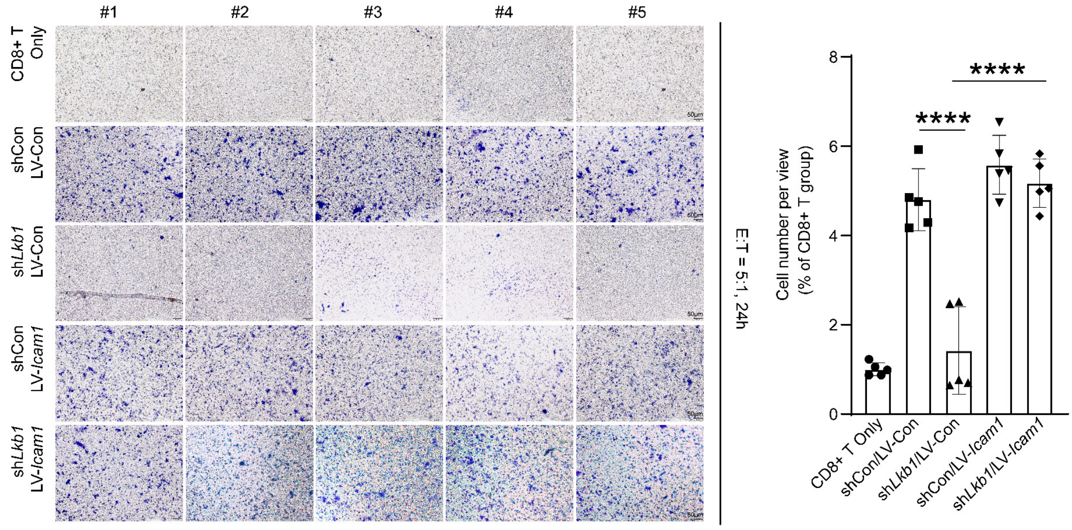

Supplementary Figure 10 (**Related with Figure 3**) T cell migration assay of LLC1 cell lines. $CD8^+$ T cells were sorted from spleen of OT-I mice and were stimulated for 5 days. LLC1 cells expressing OVA with optional expression of *LKB1* and *ICAM1* were seeded in 24-well plates and allowed to adhere overnight in complete medium. Activated $CD8^+$ T cells were then placed on the upper layer of a cell culture insert with permeable membrane for 24h. Cells that migrated to the bottom layer were stained by crystal violet blue. The E: T ratio (effector to target) was 5:1 for $CD8^+$ T cells. Representative images (left) and quantification (right) were shown. n = 5 biologically independent samples examined over 1 independent experiment. ****$p < 0.0001$, ****$p < 0.0001$. Scale bar, 50 μm (20×).

(Results are presented as mean ± SEM. One-way ANOVA followed by Tukey's multiple comparisons test was performed for multi-group comparisons. ****$p < .0001$. Source data are provided as a Source Data file.)

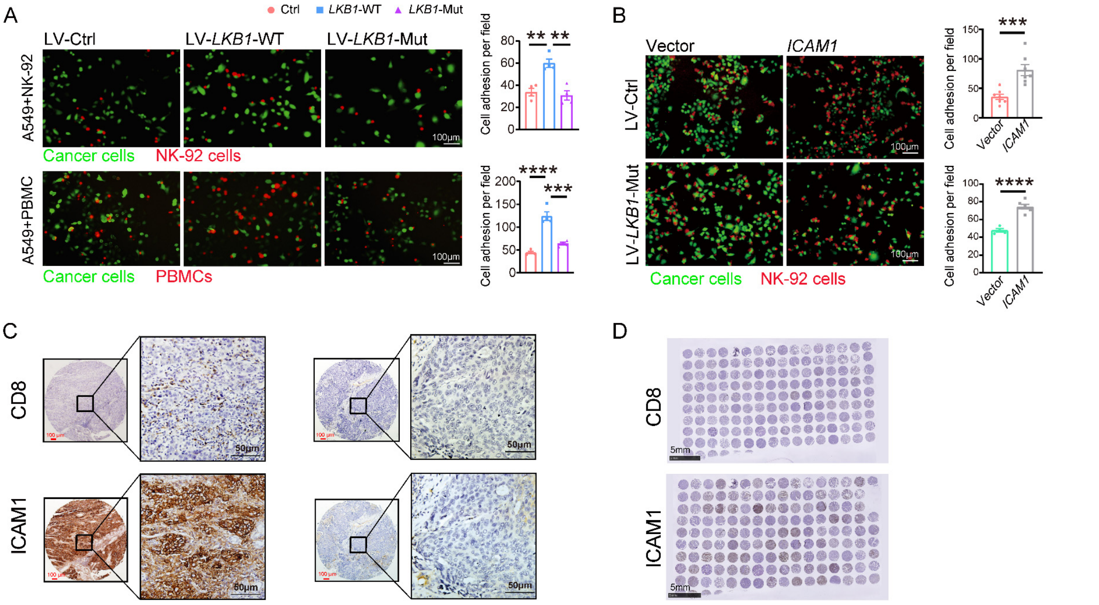

Supplementary Figure 11 (**Related with Figure 3**) ICAM1 facilitates adhesion of tumor cells to cytotoxic immune cells and promotes $CD8^+$ T cells infiltration. (A) A549 parental cells (*LKB1*-loss, referred as Ctrl) or *LKB1*-expressing A549 cells (*LKB1*-WT and *LKB1*-Mut) stained with CellTracker CFSE dye were seeded in 24-well plates and allowed to adhere overnight in complete medium. Immune cells (NK-92 cells or PBMCs) stained with CellTracker Red dye were then added to the plates containing labeled tumor cells and cocultured for 4 h before complete washing and imaging. Representative images (left) and quantification (right) were shown. n = 4 biologically independent samples examined over 1 independent experiment. **p = 0.0022, **p = 0.0011, ****p < 0.0001, ***p = 0.0001. Scale bar, 100 μm (20×). (B) Overexpression of *ICAM1* was conducted in *LKB1*-loss and *LKB1*-Mut A549 cells and adhesion assay with NK-92 cells was performed. Representative images (left) and quantification (right) were shown. In *LKB1*-loss group (Ctrl), n = 7 biologically independent samples examined over 1 independent experiment; ***p = 0.0009. In *LKB1*-Mut group, n = 4 samples in *ICAM1*-wildtpye and n = 6 in *ICAM1*-overexpressing groups; ****p < 0.0001. Scale bar, 100 μm (20×). (C-D) IHC staining of lung adenocarcinoma tissue microarray tissue sections using anti-ICAM1 and anti-CD8 antibodies. Representative images (C) and TMA snapshots (D) were shown. n = 152 biologically independent samples examined over 1 independent experiment. Scale bar, 50 μm (C, red), 100 μm (C, black). Scale bar, 5 mm (D).

(Results are presented as mean ± SEM. Two-tailed Student's t-test was used to compare the variables of two groups, and one-way ANOVA followed by Tukey's multiple comparisons test was performed for multi-group comparisons. *p < .05; **p < .01; ***p < .001; ****p < .0001. Source data are provided as a Source Data file.)

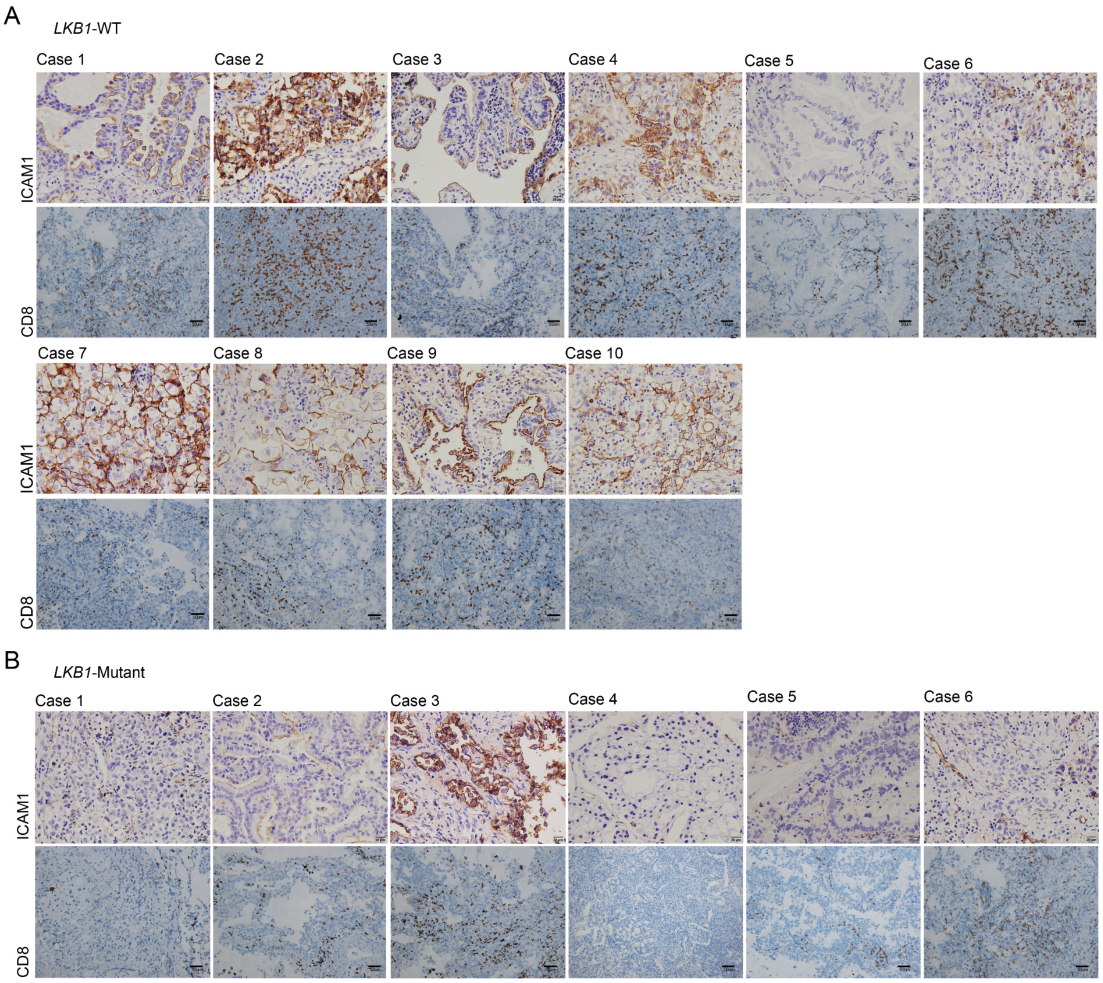

Supplementary Figure 12 (**Related with Figure 3**) Representative images of IHC staining using anti-CD8α, and ICAM1 antibodies for NSCLC patients with intact *LKB1* (A) or mutant *LKB1* (B). Scale bar, 20 μm (40×), 50 μm (20×). n = 11 biologically independent samples examined over 1 independent experiment (A). n = 7 biologically independent samples examined over 1 independent experiment (B). The representative images in Fig. 2d of IHC were specifically selected from Supplementary Figure 12.

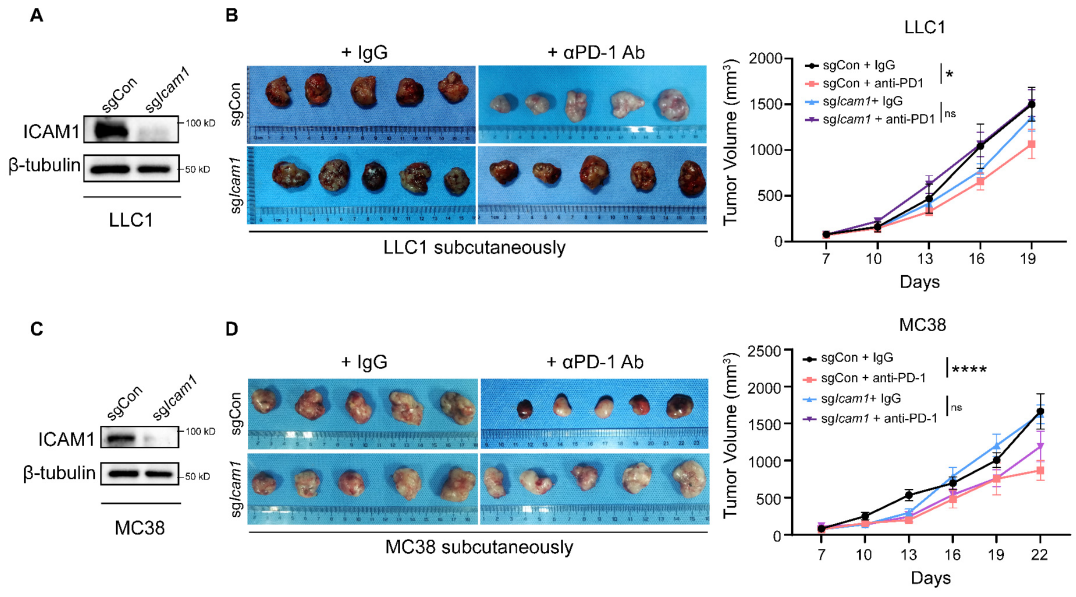

Supplementary Figure 13 (**Related with Figure 4**) Cancer cell-intrinsic knockout of *Icam1* impairs tumor responsiveness to ICB. Analysis of ICAM1 expression of LLC1 (A) and MC38 (C) cell lines transfected with sgCon or sg*Icaml* sgRNA. C57BL/6 mice were implanted subcutaneously with LLC1 or MC38 cells with optional knockout of *Icam1* and treated with or without PD-1 mAb. Macroscopic appearance of tumors (left) and plots of tumor volume (right) for LLC1 (B) and MC38 (D) xenografts. For LLC1 (B), n = 6 and n = 7 samples in sgCon receiving IgG or anti-PD-1 Ab, respectively; n = 6 and n = 5 samples in sg*Icam1* receiving IgG or anti-PD-1 Ab, respectively. A mixed-effects model followed by Tukey's multiple comparisons was performed to compare the tumor growth curves in different treatment groups. *p = 0.0259. For MC38 (D), n = 6 samples in sgCon receiving IgG or anti-PD-1 Ab; n = 7 and n = 5 samples in sg*Icam1* receiving IgG or anti-PD-1 Ab, respectively. Two-way ANOVA followed by Sidak's multiple comparisons test was performed to compare the tumor growth curves in different treatment groups. ****p = 0.0308.

(Results are presented as mean ± SEM. n.s., not significant; *p < .05; ****p < .0001. Source data are provided as a Source Data file.)

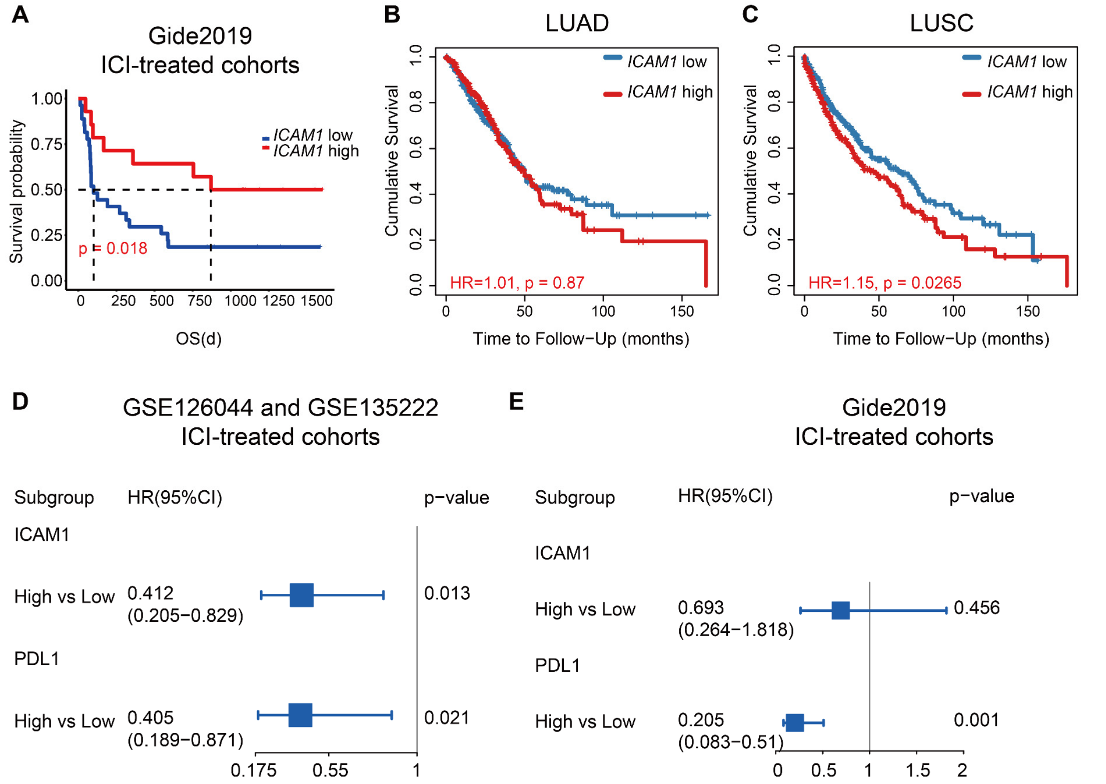

Supplementary Figure 14 (**Related with Figure 4**) Higher expression of *ICAM1* is not associated with a good prognosis but with a survival benefit in patients receiving ICI therapy. (A) Kaplan-Meier curves of progression-free survival according to the expression level of *ICAM1* in melanoma patients (Gide 2019[1], n= 41). Log-rank test was used to analyze the survival data. (B-C) Kaplan-Meier survival curves of TCGA-LUAD (B, n = 478 samples) and TCGA-LUSC (C, n = 482 samples) datasets. The survival analyses were performed using the TIMER2.0 website (http://timer.cistrome.org/). p values were calculated using the unadjusted log-rank test and hazard ratios (HRs) were calculated via a univariate Cox regression analysis. (D-E) Multivariate association of *ICAM1* and *PDL1* with progression-free survival in ICI-treated lung cancer cohort (D, GSE126044, n = 16; GSE135222, n = 27) and melanoma cohort (E, Gide 2019[1], n = 41). HRs was calculated using a multivariate Cox regression analysis. Forest plots were utilized to demonstrate the results, the error bars of which encompassed the 95% confidence intervals of HRs. Source data are provided as a Source Data file.

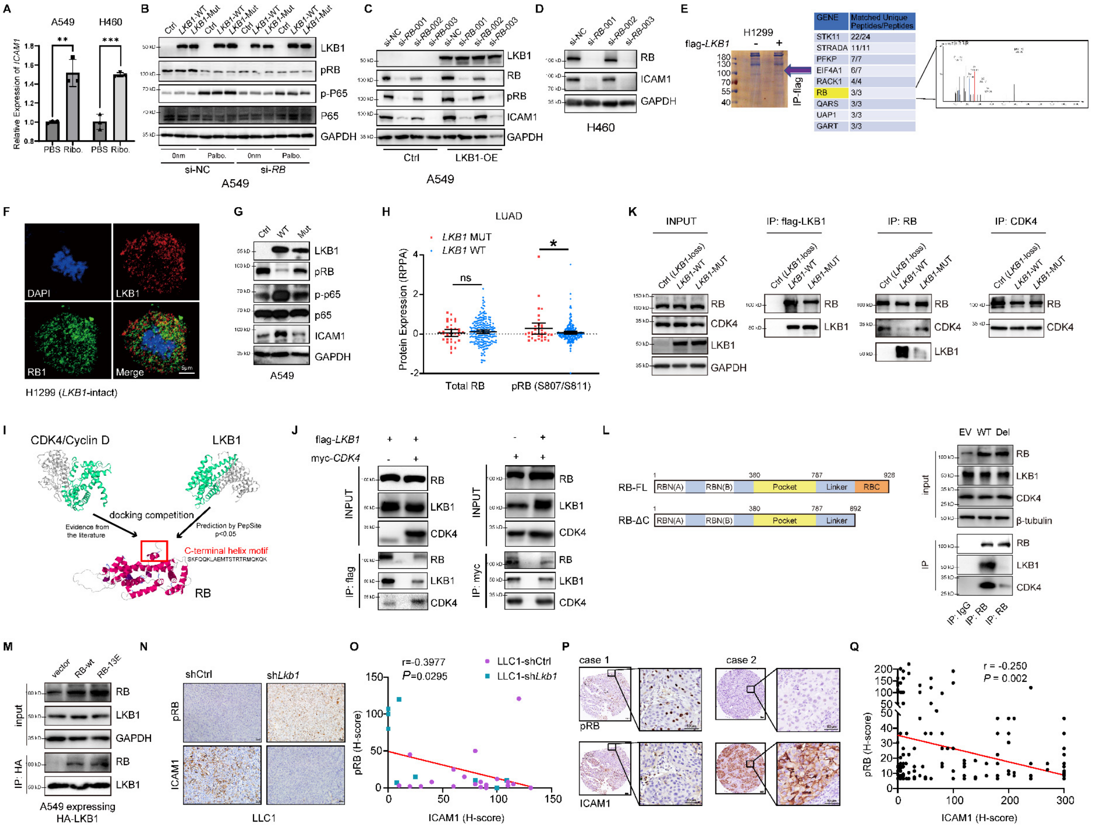

Supplementary Figure 15 (**Related with Figure 4**) Mechanistic link among LKB1, RB and CDK4. (A) RT-qPCR analysis of *ICAM1* expression in A549 and H460 cell lines after treatment of PBS or ribociclib. n = 3 biologically independent samples examined over 1 independent experiment. **p = 0.0034, ***p = 0.0005. (B) Immunoblot analysis of the indicated proteins in A549 cell lines with different *LKB1* status (wild-type, referred as *LKB1*-WT; kinase-dead mutation, referred as *LKB1*-MUT; and empty vector, referred as Ctrl) and with or without *RB* knockdown. These cells were treated with palbociclib (500 nM) or DMSO for 24 h. n = 3 independent experiments. (C) Immunoblot analysis of the indicated proteins in A549 cell lines harboring different *LKB1* status (wild-type, referred as *LKB1*-OE; empty vector, referred as Ctrl) with or without *RB* knockdown. n = 3 independent experiments. (D) Immunoblot analysis of the indicated proteins in H460 cell lines with or without *RB* knockdown. n = 3 independent experiments. (E) Immunoprecipitation (IP) analysis in H1299 cells with ectopic expression of flag-*LKB1* or transduced with parental flag-NC plasmids. n = 3 independent experiments. The result was visualized by Coomassie blue staining and further analyzed by Nano LC-ESI-MS/MS Analysis. RB was detected as the selected MS/MS spectrum showed. (F) Multiple IF labeling using LKB1 and RB antibodies in H1299 cells. n = 3 independent experiments. Scale bar, 5 μm (100×). (G) Immunoblot analysis of the indicated protein in A549 cell lines with different *LKB1* status. n = 3 independent experiments. (H) Analysis of the protein level of total RB and pRB (S807/S811) in patients with or without *LKB1* mutation using TCGA-LUAD reverse-phase protein arrays (RPPA)

dataset. n = 35 for *LKB1* MUT, n = 202 for *LKB1* WT. *p = 0.0293. (I) Bioinformatics tool (Pepsite) was utilized to predict the possibility of LKB1 docking on the C-terminal helix motif of RB (SKFQQKLAEMTSTRTRMQKQK). (J) Immunoprecipitation analysis of the relationship among LKB1, RB, and CDK4, using anti-flag or anti-myc magnetic beads in H1299 cells with or without ectopic flag-*LKB1* and myc-*CDK4* overexpression. n = 3 independent experiments. (K) Immunoprecipitation analysis of the relationship among LKB1, RB, and CDK4, using anti-LKB1 or anti-CDK4 or anti-RB antibodies in A549 cells with or without ectopic *LKB1*-WT and *LKB1*-MUT overexpression. n = 3 independent experiments. (L) RB protein variant with truncation of the RB C terminus ($Rb^{\Delta C\text{-term}}$) was constructed (RB-Del). Schematic diagram of RB truncation mutation was presented; RB-WT and RB-Del immunoprecipitate with LKB1 and CDK4, respectively. n = 3 independent experiments. (M) A549 cells expressing HA-*LKB1* were transduced with the vector, wide-type RB or phosphor-mimetic RB (13E mutant) plasmids, followed by IP analysis. n = 3 independent experiments. (N-O) IHC staining of murine xenograft tumor samples with *Lkb1* knockdown (n = 9) or with intact *Lkb1* expression (n = 21) using specific antibodies indicated. Representative images (N) and Pearson correlation analysis (O) were shown. Scale bar, 50μm. (P-Q) IHC staining of a lung adenocarcinoma tissue microarray (n = 152 TMA elements) tissue sections using anti-ICAM1 and anti-pRb antibodies. Representative images (P) and spearman correlation analysis (Q) were shown. Scale bar, 50μm.

(Results are presented as mean ± SEM. Two-tailed Student's t-test was used to analyze the data unless stated. n.s., not significant; *p < .05. Source data are provided as a Source Data file.)

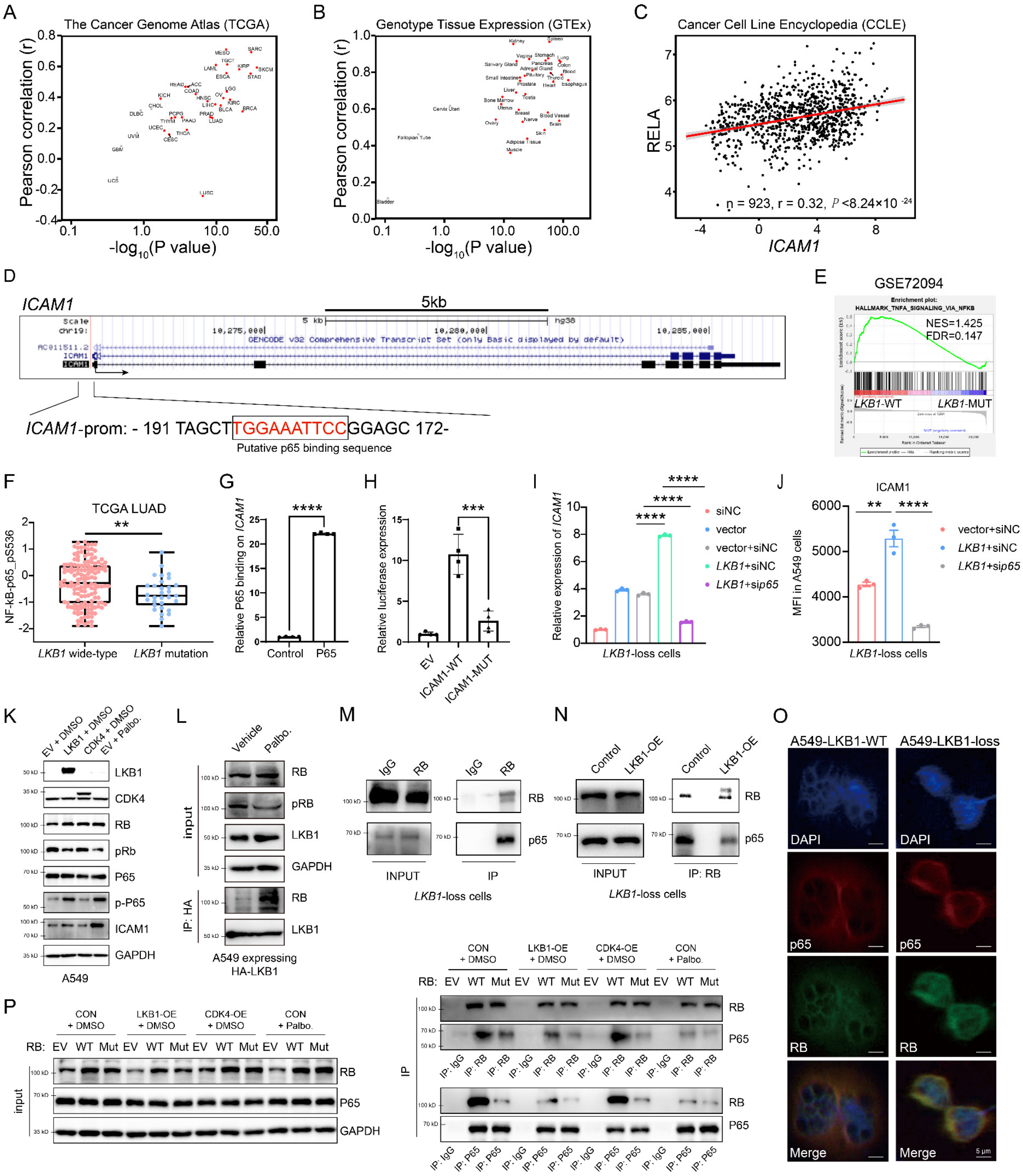

Supplementary Figure 16 (**Related with Figure 5**) (A-C) Pearson correlation analyses of *RELA* and *ICAM1* in cancer samples from The Cancer Genome Atlas database (A), normal tissues from Genotype Tissue Expression database (B), and cell lines from Cancer Cell Line Encyclopedia database (C, p < 0.0001.). Each dot represents certain cancer type (A, n = 33), tissue type (B, n = 31), and cell line (C, n = 1016). Red dots indicate $P < 0.05$ in (A) and (B). (D) The genomic loci for *ICAM1* and the putative p65 binding sequence in the promoter of *ICAM1*. (E) Gene-set enrichment analysis (GSEA) of expression profile between *LKB1*-WT group and *LKB1*-MUT group in LUAD patients (GSE72094, n = 374 samples in *LKB1*-WT group, n = 68 samples in *LKB1*-MUT group). (F) Protein level of NF-κB-p65_pS536 in LUAD patients with *LKB1* wide-type (n = 144) or *LKB1* mutation (n = 31) according to the RPPA data in TCGA. **p = 0.0079. (G) ChIP analysis of p65 binding to *ICAM1* promoter in 293T cells over-

expressing p65. n = 4 biologically independent samples examined over 1 independent experiment. ****p < 0.0001. (H) Luciferase reporter assays were conducted in the indicated cells expressing wild-type or mutated p65 binding site of *ICAM1*. n = 4 biologically independent samples examined over 1 independent experiment. ***p = 0.0001. (I-J) Analysis of ICAM1 expression of *LKB1*-loss cells expressing vector+siNC, *LKB1*+siNC, or *LKB1*+si*p65*. RT-qPCR (I) and flow cytometry (J) analysis of ICAM1 expression. n = 3 biologically independent samples examined over 1 independent experiment; ****p < 0.0001, ****p < 0.0001, ****p < 0.0001 (I). n = 3 biologically independent samples examined over 1 independent experiment; **p = 0.0015, ****p < 0.0001 (J). (K) Western blotting of certain proteins (LKB1, CDK4, RB, pRB, p65, p-p65, and ICAM1) in the vector, *LKB1*-OE, *CDK4*-OE and palbociclib groups in A549 cells. n = 3 independent experiments. (L) A549 cells expressing HA-LKB1 were treated with or without 500nM palbociclib, followed by immunoprecipitation analysis of the indicated proteins. n = 3 independent experiments. (M) Immunoprecipitation analysis of the interaction between RB and p65 performed in *LKB1*-loss cells. n = 3 independent experiments. (N) Immunoprecipitation analysis of the interaction between RB and p65 performed in A549-*LKB1*-loss cells versus A549-*LKB1*-WT cells. n = 3 independent experiments. (O) Multiple immunofluorescence labeling using anti-p65 and anti-RB antibodies in A549-*LKB1*-loss cells versus A549-*LKB1*-WT cells. n = 3 independent experiments. Scale bar, 5 μm (100×). (P) RB-WT and RB-Mut (S249A/T252A) co-immunoprecipitate with p65 in the vector, *LKB1*-overexpression, *CDK4*-overexpression and the palbociclib group. n = 3 independent experiments.

(Data were presented as box plots (median, 25%-75%, range) or mean ± SEM. Two-tailed Student's t-test was used to compare the variables of two groups, and one-way ANOVA followed by Tukey's multiple comparisons test was performed for multi-group comparisons. **p < .01; ***p < .001; ****p < .0001. Source data are provided as a Source Data file.)

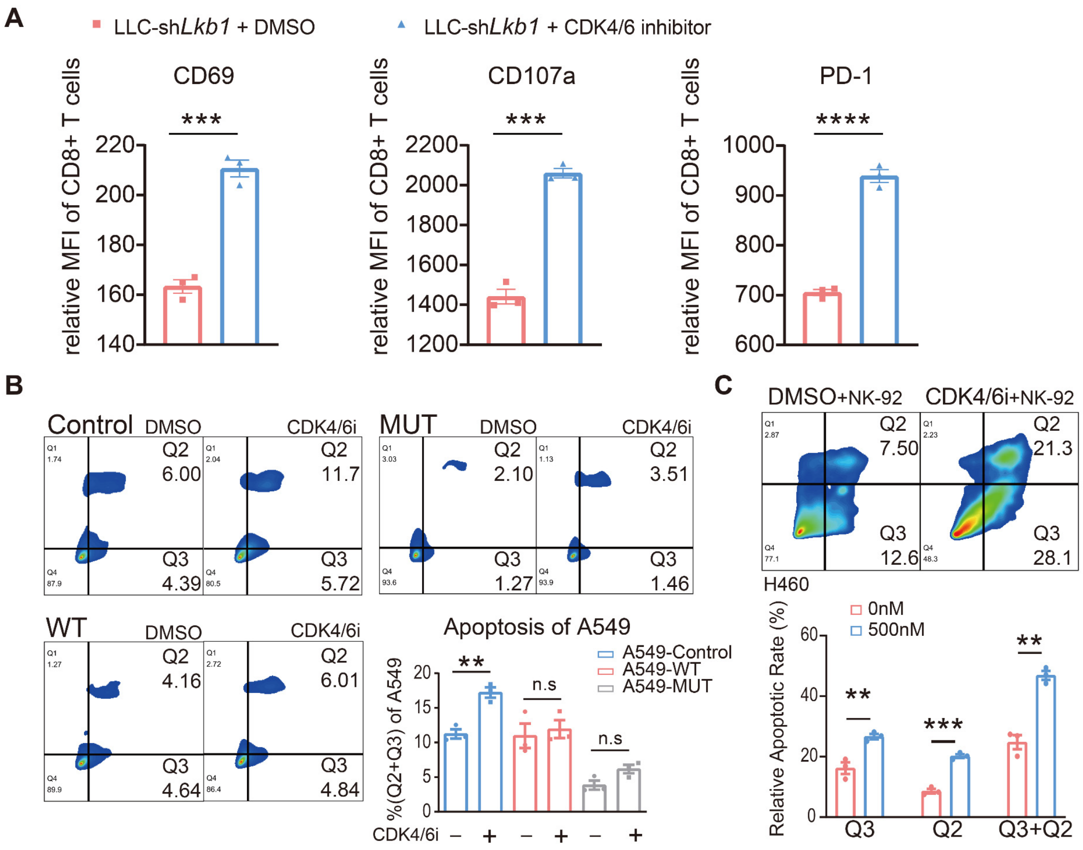

Supplementary Figure 17 (**Related with Figure 5**) CDK4/6 inhibition on tumor cells reinforced their interactions with T cells and NK cells. (A) LLC1-sh*Lkb1* tumor cells were pre-treated with CDK4/6 inhibitors or DMSO for 48 hours, and subsequently co-cultured with activated CD8^{+} T cells for another 24 hours. T cells were subsequently sorted and analyzed with flow cytometry. E: T = 5:1. n = 3 biologically independent samples examined over 1 independent experiment. ***p = 0.0004, ***p = 0.0001, ****p < 0.0001. (B) A549 cells with different *LKB1* status (wild-type, referred as WT; kinase-dead mutation, referred as MUT; and empty vector, referred as Control) were treated with CDK4/6 inhibitors or DMSO, and analyzed apoptosis by flow cytometry. n = 3 biologically independent samples examined over 1 independent experiment. **p = 0.0039. (C) H460 cells were pre-treated with CDK4/6 inhibitors or DMSO, and subsequently co-cultured with NK-92 cells before assessment of apoptosis by flow cytometry. Representative images and quantification results were shown. n = 3 biologically independent samples examined over 1 independent experiment. **p = 0.0075, ***p = 0.0003, **p = 0.0014.

(Results are presented as mean ± SEM. Two-tailed Student's t-test was used to compare the variables of two groups. n.s., not significant; **p < .01; ***p < .001; ****p < .0001. Source data are provided as a Source Data file.)

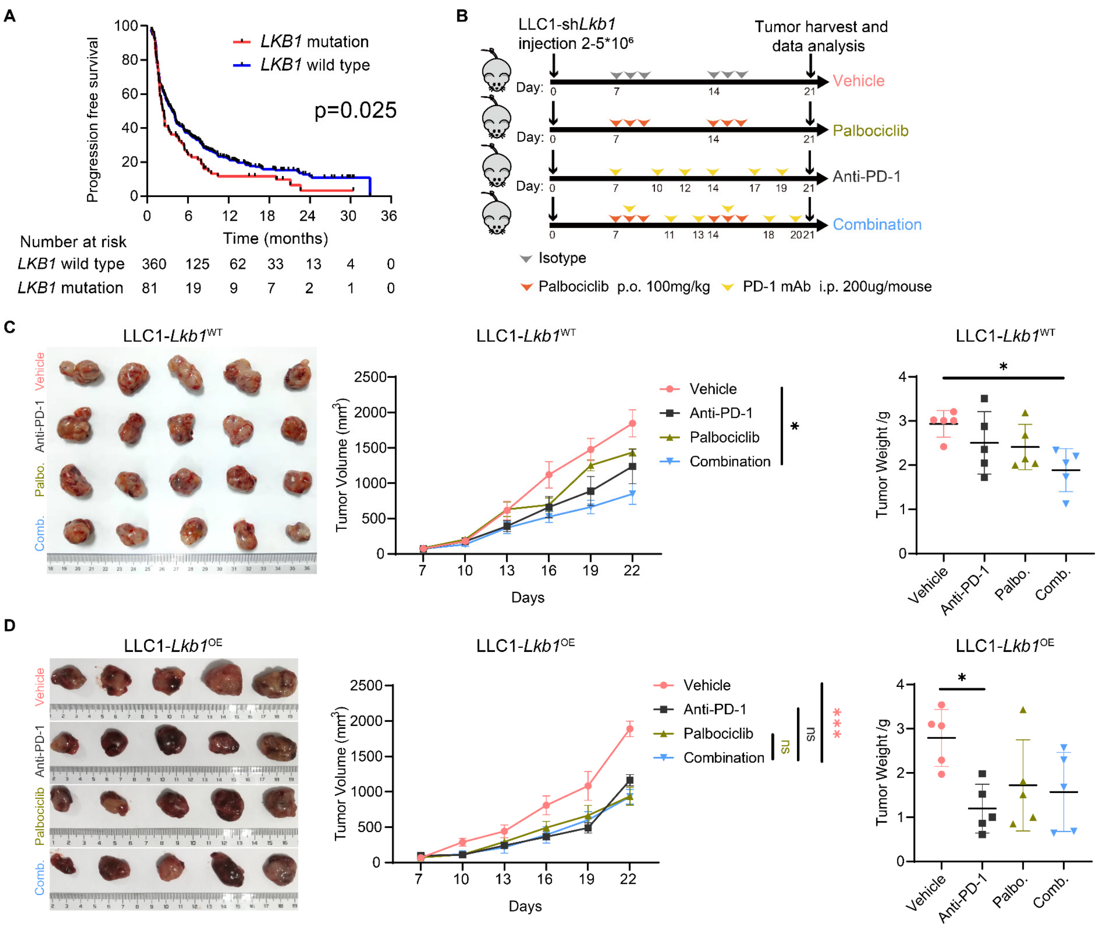

Supplementary Figure 18 (Related with Figure 6) CDK4/6 inhibitors sensitize *LKB1*-deficient tumors to ICI therapy. (A) Kaplan-Meier curves of progression-free survival according to *LKB1* status in non-small cell lung cancer (NSCLC) patients. n = 81 samples in *LKB1* mutation group, n = 360 samples in *LKB1* wild type group. The p value was calculated using the log-rank test. (B) A schematic representation of the treatment plan. (C-D) C57BL/6 mice were implanted subcutaneously with LLC1 cell lines with wild-type *Lkb1* (C) or overexpressed *Lkb1* (D) and treated with isotype IgG, anti-PD-1 Ab, palbociclib or combination of both anti-PD-1 Ab and palbociclib. Macroscopic appearance of tumors (left), plots of tumor volume (middle) and quantification of tumor weight (right) were shown. n = 5 biologically independent mice examined over 1 independent experiment; *p = 0.0157, *p = 0.0273 (C). n = 5 biologically independent mice examined over 1 independent experiment; ***p = 0.001, *p = 0.0284 (D). (Results are presented as mean ± SEM. Two-way ANOVA followed by Tukey's multiple comparisons was performed to compare the tumor growth curves in different treatment groups, and one-way ANOVA was performed for multi-group comparisons. n.s., not significant; *p < .05; ***p < .001. Source data are provided as a Source Data file.)

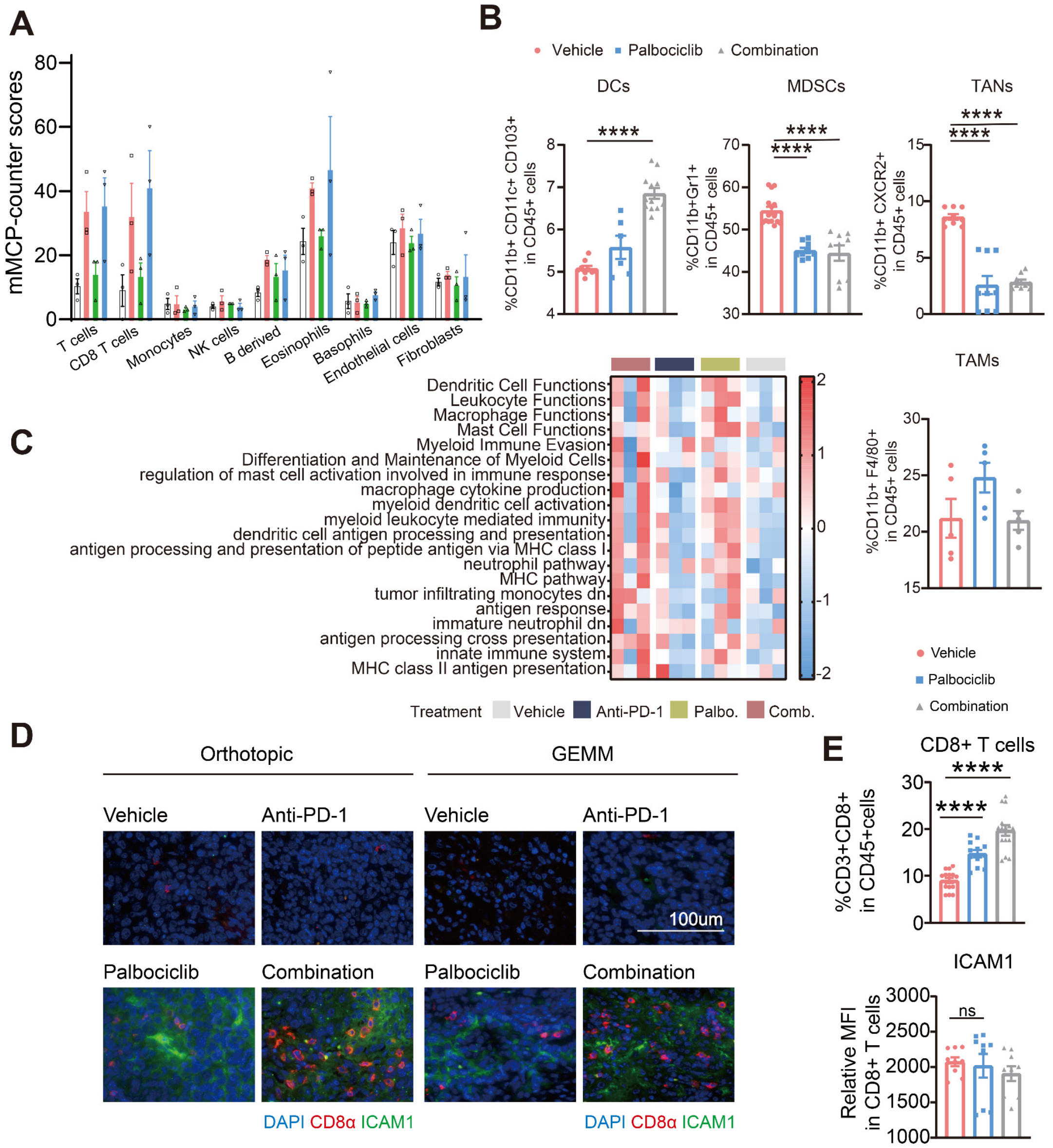

Supplementary Figure 19 (**Related with Figure 7**) Combined CDK4/6 inhibitors and PD-1 blockade therapy may affect myeloid cells and CD8$^+$ T cells as well. (A) Quantification of murine microenvironment cell population counter (mMCPcounter) analysis of immune cell infiltration levels in different treatment arms. n = 3 biologically independent samples. (B) Flow cytometry analysis of infiltration of DCs (CD45$^+$ CD11b$^+$ CD11c$^+$ CD103$^+$), MDSCs (CD45$^+$ CD11b$^+$ Gr1$^+$), TAN (CD45$^+$ CD11b$^+$ CXCR2$^+$), and TAMs (CD45$^+$ CD11b$^+$ F4/80$^+$) in LLC1-sh*Lkb1* tumors with different treatments. DCs, n = 9 for vehicle, n = 6 for palbociclib, and n = 12 for combination; ****p < 0.0001. MDSCs, n = 15 for vehicle, n = 9 for palbociclib, and n = 9 for combination; ****p < 0.0001, ****p < 0.0001. TANs, n = 9 biologically independent samples examined over 1 independent experiment; ****p < 0.0001, ****p < 0.0001. TAMs, n = 5 biologically independent samples examined over 1 independent experiment. (C) Gene set variation analysis of mRNA sequencing data of tumor tissue in four treatment arms with gene sets related to the

function of myeloid immune cells from the nCounter PanCancer Immune Profiling Panel and the Molecular Signatures Database. n = 3 biologically independent samples. (D) Immunofluorescence (IF) analysis of ICAM1$^+$ cells (green), CD8$^+$ T cells (red) in resected KL GEMM samples (right) and orthotopic mouse models (left) following different arms of treatment. n = 5 biologically independent mice examined over 1 independent experiment. Scale bar, 100 µm. (E) Flow cytometry analysis of infiltration of CD8$^+$ T cells and their ICAM1 membrane expression in LLC1-sh*Lkb1* tumors with different treatments. CD8$^+$ T cells, n = 15 for vehicle, n = 12 for palbociclib, and n = 15 for combination; ****p < 0.0001, ****p < 0.0001. ICAM1, n = 9 biologically independent samples examined over 1 independent experiment.

(Results are presented as mean ± SEM. One-way ANOVA followed by Tukey's multiple comparisons test was performed for multi-group comparisons. n.s., not significant; ****p < .0001. Source data are provided as a Source Data file.)

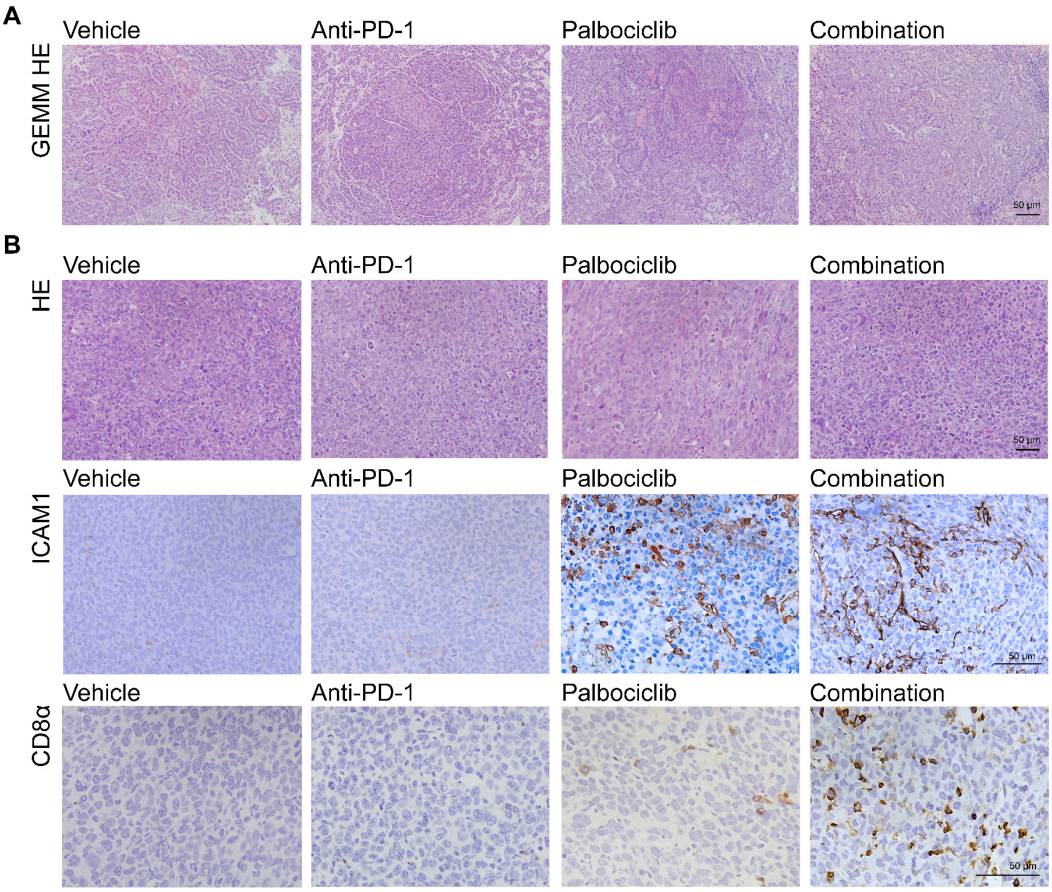

Supplementary Figure 20 (Related with Figure 8) Combination therapy induces a favorable TME. (A) Representative HE staining of resected tumor samples in GEMM KL mouse administered to indicated treatment. n = 5 biologically independent samples examined over 1 independent experiment. Scale bar, 50 µm. (B) Representative HE staining and IHC images of ICAM1 and CD8α in orthotopic lung tumors subjected to indicated treatment. n = 5 biologically independent samples examined over 1 independent experiment. Scale bar, 50 µm.

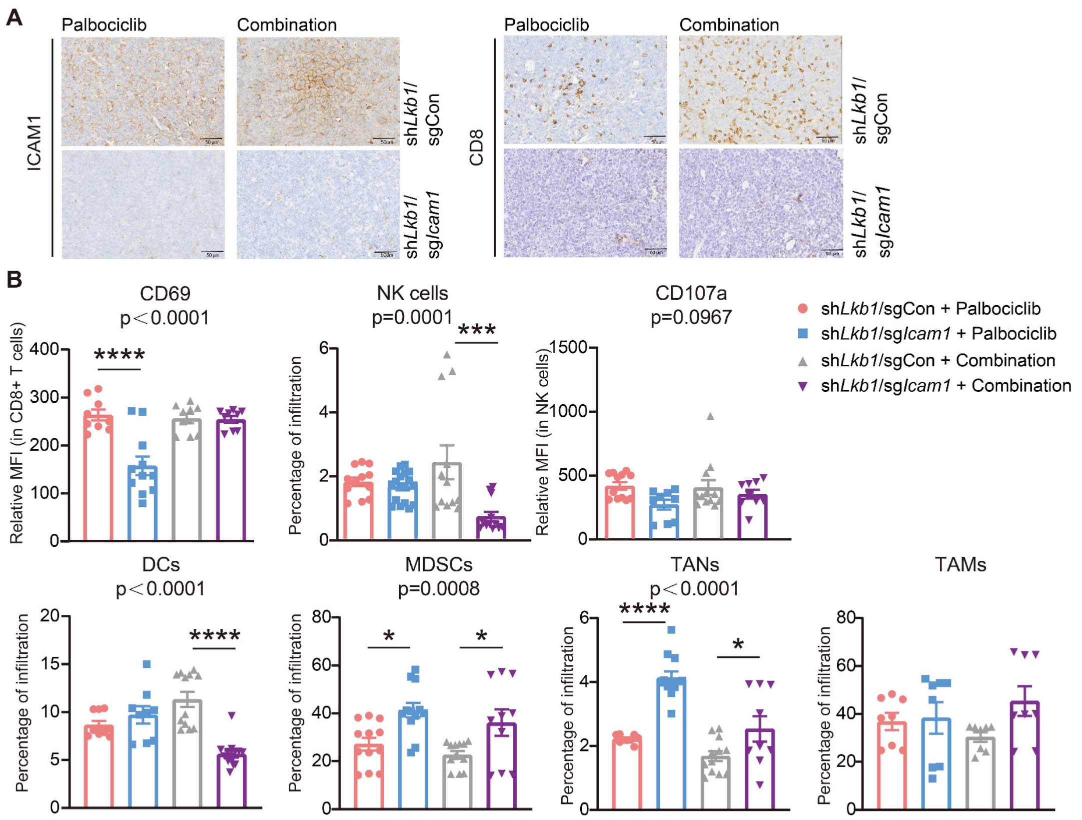

Supplementary Figure 21 (**Related with Figure 8**) ICAM-1 depletion abrogates anti-tumor immunity conferred by the palbociclib/ICI combination therapy. (**A**) Representative IHC images of ICAM1 and CD8α in LLC1-sh*Lkb1*/sgCon and LLC1-sh*Lkb1*/sg*Icam1* tumors subjected to indicated treatment. n = 5 biologically independent samples examined over 1 independent experiment. Scale bar, 50 μm. (**B**) Flow cytometry analysis of immune cell infiltration, expression of CD69 on CD8$^+$ T cells and CD107a on NK cells in sh*Lkb1*/sgCon and sh*Lkb1*/sg*Icam1* tumors followed by indicated treatment arm. CD69, n = 9, n = 11, n = 8, and n = 9 in sgCon+palbociclib, sg*Icam1*+palbociclib, sgCon+combination and sg*Icam1*+combination groups, respectively; ****p < 0.0001. NK cells, n = 12, n = 18, n = 12, and n = 12 in sgCon+palbociclib, sg*Icam1*+palbociclib, sgCon+combination and sg*Icam1*+combination groups, respectively; ***p = 0.0004. CD107a, n = 12, n = 9, n = 12, and n = 9 in sgCon+palbociclib, sg*Icam1*+palbociclib, sgCon+combination and sg*Icam1*+combination groups, respectively. DCs, n = 9, n = 9, n = 12, and n = 14 in sgCon+palbociclib, sg*Icam1*+palbociclib, sgCon+combination and sg*Icam1*+combination groups, respectively; ****p < 0.0001. MDSCs, n = 12, n = 12, n = 12, and n = 10 in sgCon+palbociclib, sg*Icam1*+palbociclib, sgCon+combination and sg*Icam1*+combination groups, respectively; *p = 0.0167, *p = 0.0364. TANs, n = 9, n = 12, n = 12, and n = 9 in sgCon+palbociclib, sg*Icam1*+palbociclib, sgCon+combination and sg*Icam1*+combination groups, respectively; ****p < 0.0001. TAMs, n = 8 samples in each group.

(Results are presented as mean ± SEM. One-way ANOVA followed by Tukey's multiple comparisons test was performed for multi-group comparisons. $*p < .05$; $***p < .001$; $****p < .0001$. Source data are provided as a Source Data file.)

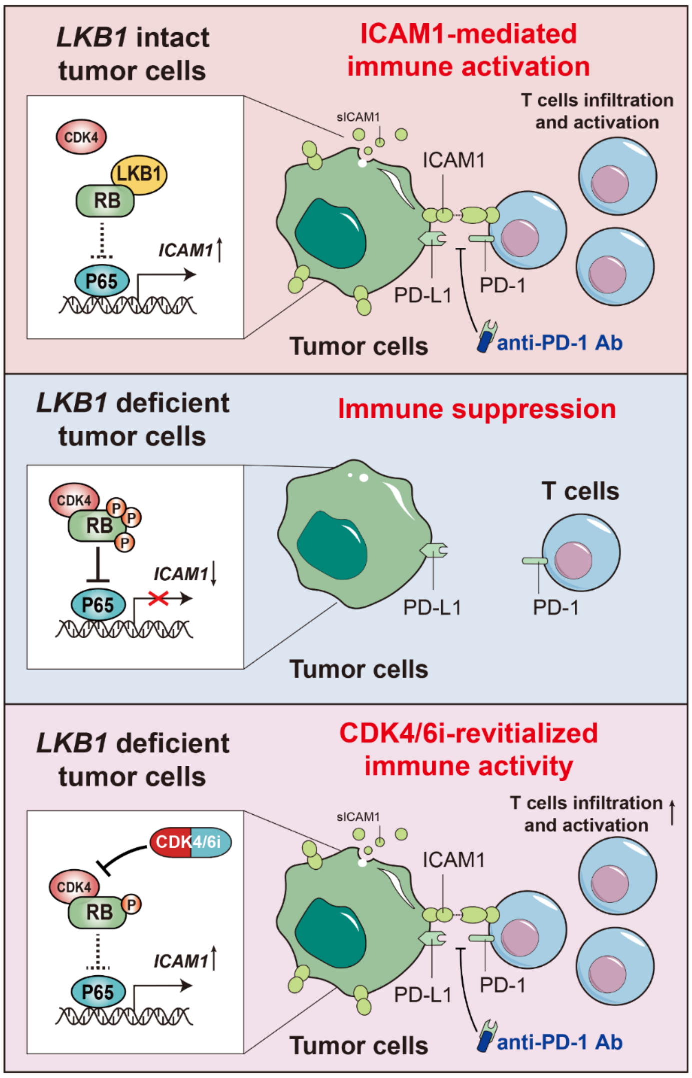

Supplementary Figure 22 (Related with Figure 8) Schematic of the mechanism through which mutant *LKB1* downregulates *ICAM1* transcription and expression in a phosphorylated RB-dependent manner, and leads to impaired cancer cell-T cell adhesion and interaction. CDK4/6 inhibitors activate *ICAM1* transcription, improve cytotoxic cell infiltration and activity, and re-sensitize *LKB1* mutant tumors to anti-PD-1 immunotherapy.

References:

1. Gide TN, *et al.* Distinct Immune Cell Populations Define Response to Anti-PD-1 Monotherapy and Anti-PD-1/Anti-CTLA-4 Combined Therapy. *Cancer Cell* **35**, 238-255 e236 (2019).
