# Supplementary material for: CDK4/6 inhibition triggers ICAM1-driven immune response and sensitizes LKB1 mutant lung cancer to immunotherapy
Source: Nat Commun. 2023 Mar 4;14:1247. doi: 10.1038/s41467-023-36892-4 (PMC9985635; doi:10.1038/s41467-023-36892-4)
Supplement: Supplementary file 3 — Description of Additional Supplementary Files [file 41467_2023_36892_MOESM3_ESM.pdf]

Description of Additional Supplementary Files:

File Name: Supplementary Data 1
Description: Cell markers used for specific cell clusters

File Name: Supplementary Data 2
Description: Communication signatures for 11 cell types identified in the scRNA data

File Name: Supplementary Data 3
Description: qPCR primers

File Name: Supplementary Data 4
Description: Antibodies
