# Supplementary material for: CDK4/6 inhibition triggers ICAM1-driven immune response and sensitizes LKB1 mutant lung cancer to immunotherapy
Source: Nat Commun. 2023 Mar 4;14:1247. doi: 10.1038/s41467-023-36892-4 (PMC9985635; doi:10.1038/s41467-023-36892-4)
Supplement: Supplementary file 8 — Reporting Summary [file 41467_2023_36892_MOESM8_ESM.pdf]

nature portfolio

Corresponding author(s): Zhong-Yi Dong

Last updated by author(s): Feb 17, 2023

Reporting Summary

Nature Portfolio wishes to improve the reproducibility of the work that we publish. This form provides structure for consistency and transparency in reporting. For further information on Nature Portfolio policies, see our Editorial Policies and the Editorial Policy Checklist.

Statistics

For all statistical analyses, confirm that the following items are present in the figure legend, table legend, main text, or Methods section.

n/a	Confirmed	
☐	☒	The exact sample size (n) for each experimental group/condition, given as a discrete number and unit of measurement
☐	☒	A statement on whether measurements were taken from distinct samples or whether the same sample was measured repeatedly
☐	☒	The statistical test(s) used AND whether they are one- or two-sided<br>*Only common tests should be described solely by name; describe more complex techniques in the Methods section.*
☒	☐	A description of all covariates tested
☐	☒	A description of any assumptions or corrections, such as tests of normality and adjustment for multiple comparisons
☐	☒	A full description of the statistical parameters including central tendency (e.g. means) or other basic estimates (e.g. regression coefficient) AND variation (e.g. standard deviation) or associated estimates of uncertainty (e.g. confidence intervals)
☐	☒	For null hypothesis testing, the test statistic (e.g. F, t, r) with confidence intervals, effect sizes, degrees of freedom and P value noted<br>*Give P values as exact values whenever suitable.*
☒	☐	For Bayesian analysis, information on the choice of priors and Markov chain Monte Carlo settings
☒	☐	For hierarchical and complex designs, identification of the appropriate level for tests and full reporting of outcomes
☐	☒	Estimates of effect sizes (e.g. Cohen's d, Pearson's r), indicating how they were calculated

Our web collection on statistics for biologists contains articles on many of the points above.

Software and code

Policy information about availability of computer code

Data collection	Roche LightCycler 480 System; BD LSR II or LSRFortessa flow cytometer; Illumina Novaseq platform; Illumina Xten platform; AMI HTX imaging system (Spectral Instruments Imaging); MRI (PharmaScan70/16 US); micro-CT scanner (PINGSENG Healthcare); Ekspert NanoLC 415 system (SCIEX, Concord, ON); Triple TOF 6600 tandem mass spectrometer (Sciex, Concord, Ontario, Canada); 3-D slicer software (version 5.0.2).
Data analysis	FlowJo software (version 10.5; Tree Star); Graphpad Prism (v8); R-4.1.0; Single cell sequencing data analysis: trimomatic software (Version 0.39), CellRanger (version 6.0, 10x Genomics), Seurat (R package, version 3.1.5), CellPhone DB (v2.0.0); BiomaRt (R package, v2.54.0); clusterProfiler (R package, v4.6.0); GAGE (R package, version 2.22.0); GSVA (R package, v1.40.1).

For manuscripts utilizing custom algorithms or software that are central to the research but not yet described in published literature, software must be made available to editors and reviewers. We strongly encourage code deposition in a community repository (e.g. GitHub). See the Nature Portfolio guidelines for submitting code & software for further information.

Data

Policy information about availability of data

All manuscripts must include a data availability statement. This statement should provide the following information, where applicable:

- Accession codes, unique identifiers, or web links for publicly available datasets
- A description of any restrictions on data availability
- For clinical datasets or third party data, please ensure that the statement adheres to our policy

Both raw and processed RNA-seq data for single cell RNA sequencing and murine tumors treated by different therapeutic panels have been deposited in NCBI's

Gene Expression Omnibus and are accessible through accession number GEO: GSE18096 (https://www.ncbi.nlm.nih.gov/geo/query/acc.cgi?acc=GSE180963), and GSE182228 (https://www.ncbi.nlm.nih.gov/geo/query/acc.cgi?acc=GSE182228). The publicly available data used in this study are available in the GEO database or the European Nucleotide Archive (ENA) under accession code GSE72094(https://www.ncbi.nlm.nih.gov/geo/query/acc.cgi?acc=GSE72094), GSE126044(https://www.ncbi.nlm.nih.gov/geo/query/acc.cgi?acc=GSE126044). GSE135222(https://www.ncbi.nlm.nih.gov/geo/query/acc.cgi?acc=GSE135222), GSE93157(https://www.ncbi.nlm.nih.gov/geo/query/acc.cgi?acc=GSE93157), GSE110397(https://www.ncbi.nlm.nih.gov/geo/query/acc.cgi?acc=GSE110397), GSE137244(https://www.ncbi.nlm.nih.gov/geo/query/acc.cgi?acc=GSE137244), GSE110397(https://www.ncbi.nlm.nih.gov/geo/query/acc.cgi?acc=GSE110397) and PRJEB23709 (https://www.ebi.ac.uk/ena/browser/view/PRJEB23709). The RNA-seq TPM matrix of Liu's melanoma immunotherapy cohort can be download from https://www.nature.com/articles/s41591-019-0654-5#additional-information. mm10 reference genome used in the manuscript was downloaded from http://hgdownload.cse.ucsc.edu/goldenPath/mm10/bigZips/chromFaMasked.tar.gz. Source data are provided with this paper in a separate Excel file.

Human research participants

Policy information about studies involving human research participants and Sex and Gender in Research.

Reporting on sex and gender	not aplicable
Population characteristics	not aplicable
Recruitment	not aplicable
Ethics oversight	not aplicable

Note that full information on the approval of the study protocol must also be provided in the manuscript.

Field-specific reporting

Please select the one below that is the best fit for your research. If you are not sure, read the appropriate sections before making your selection.

[x] Life sciences [ ] Behavioural & social sciences [ ] Ecological, evolutionary & environmental sciences

For a reference copy of the document with all sections, see nature.com/documents/nr-reporting-summary-flat.pdf

Life sciences study design

All studies must disclose on these points even when the disclosure is negative.

Sample size	Sample sizes were determine based on our previous experience with these experiments, and no statistical method was used to predetermined sample size. Instead, For assays where variability is commonly high, we used n=6. For assays with low variability, we typically used n=3.
Data exclusions	No data was excluded from analysis. All dots plots in this study show all data points from min to max.
Replication	All data was reproduced by either biological replicates or independent technical replicates at least 3 times, with the exception of fig.5f.
Randomization	For all the in vivo experiments, the animals were randomly distributed and assigned to different treatment groups prior to the start of the treatment. For all in vitro experiments, cells were seeded into plates randomly.
Blinding	For all in vivo experiments, all the analyzing data were scored blindly. For in vitro experiments, images were scored blindly. For all FACS assays, no blinding took place as sample name and condition were necessary to perform and analyze the FACS experiment.

Reporting for specific materials, systems and methods

We require information from authors about some types of materials, experimental systems and methods used in many studies. Here, indicate whether each material, system or method listed is relevant to your study. If you are not sure if a list item applies to your research, read the appropriate section before selecting a response.

Materials & experimental systems

n/a	Involved in the study
	x Antibodies
	x Eukaryotic cell lines
x	Palaeontology and archaeology
	x Animals and other organisms
x	Clinical data
x	Dual use research of concern

Methods

n/a	Involved in the study
x	ChIP-seq
	x Flow cytometry
x	MRI-based neuroimaging

Antibodies

Antibodies used

LKB1, Rabbit monoclonal, clone 27D10, 1/1000 WB, 1/100 IP, CST, #3050
LKB1, Rabbit monoclonal, clone D60C5F10, 1/250 IHC, CST, #13031
LKB1, Mouse monoclonal, clone 1H8E4, 1/200 IF, Proteintech, #66719-1-Ig
anti-mouse CD8α, Rabbit monoclonal, clone D4W2Z, 1/200 IHC, CST, #98941
anti-human CD8α, Mouse monoclonal, clone rC8/468, 1/500 IHC, Abcam, #237941
anti-mouse CD8α, Rabbit monoclonal, clone EPR21769, 1/200 IF, Abcam, #217344
AMPKα, Rabbit monoclonal, clone D5A2, 1/1000 WB, CST, #5831
Phospho-AMPKα (Thr172), Rabbit polyclonal, 1/1000 WB, CST, #2531
NF-κB p65, Rabbit monoclonal, clone D14E12, 1/1000 WB, 1/100 CHIP, CST, #8242
NF-κB p65, Mouse monoclonal, clone L8F6, 1/1000 IF, CST, #6956
Phospho-NF-κB p65 (Ser536), Rabbit monoclonal, clone 93H1, 1/1000 WB, 1/500 IHC, CST, #3033
Rb, Rabbit monoclonal, clone D20, 1/1000 WB, 1/100 IP, CST, #9313
Rb, Rabbit monoclonal, clone EPR17512, 1/500 IF, Abcam, #181616
Phospho-Rb (Ser807/811), Rabbit monoclonal, clone D20B12, 1/3000 WB, 1/500 IF, 1/500 IHC, CST, #8516
anti-human ICAM-1, Rabbit monoclonal, clone E3Q9N, 1/1000 WB, 1/200 IHC, CST, #67836
anti-mouse ICAM-1, Rabbit monoclonal, clone EPR16608, 1/1000 WB, 1/2000 IHC, Abcam, #179707
anti-mouse ICAM-1, Mouse monoclonal, clone 2F9A8, 1/50 IF, Proteintech, #60299
CDK4, Mouse monoclonal, clone 1G2C12, 1/1000 WB, Proteintech, #66950-1-Ig
CDK4, Rabbit monoclonal, clone EPR17525, 1/50 IP, Abcam, #199728
GAPDH, Mouse monoclonal, 1/10000 WB, DIA-AN, #2058
β-Actin, Rabbit monoclonal, 1/5000 WB, CST, #4970
β-Tubulin, Rabbit monoclonal , 1/5000 WB, CST, #2146
MYC-Tag, Mouse monoclonal, 1/100 IP, DIA-AN, #2097
FLAG-Tag, Mouse monoclonal, 1/100 IP, DIA-AN, #2064
HA-Tag, Mouse monoclonal, 1/100 IP, DIA-AN, #2063

PerCP/Cyanine5.5 anti-mouse CD45, Rat monoclonal, 30-F11, 1/100 FC, Biolegend, #103131
Brilliant Violet 605™ anti-mouse CD45, Rat monoclonal, 30-F11, 1/100 FC, Biolegend, #103140
PerCP/Cyanine5.5 anti-mouse CD3, Rat monoclonal, 17A2, 1/100 FC, Biolegend, #100217
FITC anti-mouse CD8a, Rat monoclonal, 53-6.7, 1/200 FC, Biolegend, #100705
APC/Cyanine7 anti-mouse CD69, Armenian hamster monoclonal, H1.2F3, 1/100 FC, Biolegend, #104525
PE/Cyanine7 anti-mouse CD279 (PD-1), Rat monoclonal, RMP1-30, 1/100 FC, Biolegend, #109110
Brilliant Violet 421™ anti-mouse/human CD44, Rat monoclonal, IM7, 1/25 FC, Biolegend, #103040
PE/Cyanine7 anti-mouse CD182 (CXCR2), Rat monoclonal, SA044G4, 3/100 FC, Biolegend, #149315
PE anti-human CD54, Mouse monoclonal, HA58, 1/100 FC, Biolegend, #353106
Brilliant Violet 421™ anti-mouse F4/80, Rat monoclonal, BM8, 1/100 FC, Biolegend, #123132
Brilliant Violet 510™ anti-mouse CD62L, Rat monoclonal, MEL-14, 1/25 FC, Biolegend, #104441
PE/Dazzle™ 594 anti-mouse Ki-67, Rat monoclonal, 16A8, 3/100 FC, Biolegend, #652428
Brilliant Violet 510™ anti-mouse/human CD11b, Rat monoclonal, M1/70, 1/100 FC, Biolegend, #101263
APC anti-mouse CD107a (LAMP-1), Rat monoclonal, 1D4B, 1/50 FC, Biolegend, #121614
Brilliant Violet 510™ anti-mouse IFN-γ, Rat monoclonal, XMG1.2, 1/200 FC, Biolegend, #505841
PE anti-mouse NK-1.1, Mouse monoclonal, S17016D, 1/100 FC, Biolegend, #156503
APC anti-mouse CD274 (B7-H1, PD-L1), Rat monoclonal, 10F.9G2, 1/400 FC, Biolegend, #124311
FITC anti-mouse CD326 (Ep-CAM), Rat monoclonal, G8.8, 1/100 FC, Biolegend, #118207
APC anti-mouse Ly-6G/Ly-6C (Gr-1), Rat monoclonal, RB6-8C5, 1/200 FC, Biolegend, #108411
PE anti-mouse CD11c, Armenian hamster monoclonal, N418, 1/400 FC, Biolegend, #117307
FITC anti-mouse CD103, Rat monoclonal, W19396D, 1/400 FC, Biolegend, #110907

anti-mouse LFA-1α (CD11a), clone FD441.8, 1/100, in vitro blockade, BioXCell, #BE0005-1
anti-mouse/human CD11b, clone M1/70, 1/100, in vitro blockade, BioXCell, #BE0007
anti-mouse CD8α, clone 2.43, 200 μg/mouse, in vivo, BioXCell, #BE0061
anti-mouse NK1.1, clone PK136, 250 μg/mouse, in vivo, BioXCell, #BE0036
anti-mouse CD54 (ICAM-1), clone YN1/1.7.4, 200 μg/mouse, in vivo, BioXcell, #BE0020-1
anti-mouse PD-1 (CD279), clone RMP1-14, 200 μg/mouse, in vivo, BioXCell, #BE0146

	rat IgG2a isotype control, clone 2A3, BioXcell, #BP0089
Validation	All antibodies used in this study are commercially available, and product information is readily available at the manufacturer's website.

Eukaryotic cell lines

Policy information about cell lines and Sex and Gender in Research

Cell line source(s)	H460, H1299, A549, H1573, H1944, Calu1, H3255, and HCC4006 cells were provided by Guangdong Lung Cancer Institute, who purchased these cells from the American Type Culture Collection (ATCC), and Authentication of Human Cell Lines Reports of these cell lines were provided. All of them were matched with alleles of corresponding cells from ATCC. LLC1 cell line was purchased from Guang Zhou Jennio Biotech Co.,Ltd. NK-92 cells were provided by Chou Yang, who purchased cells from Guang Zhou Jennio Biotech Co.,Ltd. All cells were maintained in a humidified incubator at 37°C with 5% CO2, and grown in RPMI 1640 or DMEM supplemented with 10% FBS and 100 IU/ml penicillin/streptomycin. All cell lines used were negative for mycoplasma. An updated detection report of mycoplasma was also provided.
Authentication	Cell lines were authenticated.
Mycoplasma contamination	All cell lines used tested negative for mycoplasma.
Commonly misidentified lines (See ICLAC register)	This study did not use commonly misidentified lines

Animals and other research organisms

Policy information about studies involving animals; ARRIVE guidelines recommended for reporting animal research, and Sex and Gender in Research

Laboratory animals	C57BL/6 mice (5–8 weeks old) :Both males and females were used. All mice were between 6-8 weeks old at the start of each experiment.<br>KrasG12D/+ mice: Both males and females were used. When the mice were 4-6 weeks old, lentiviruses were given by nasal inhalation. Tumor formation was analyzed with an MRI (PharmaScan70/16 US) or micro-CT scanner (PINGSENG Healthcare) after 12 weeks of virus infection. Once we had observed gasping from lung tumor-burdened mice and monitored lung nodules, then these mice were randomized into various study cohorts.
Wild animals	This study did not involve wild animals.
Reporting on sex	The findings apply to both sexes. Both male and female mice were used in this study. No selection for sex of mice was performed. As the frequency of male and female STK11-mut patients is similar in the clinical setting, as demonstrated by previous studies (doi: 10.1158/2159-8290.CD-18-0099; 10.1097/JTO.0000000000000391), we did not take sex into consideration. Furthermore, the immune phenotype and molecular mechanism of STK11-mut tumors are not influenced by gender.
Field-collected samples	This study did not involve samples collected from the field
Ethics oversight	The study protocol of mouse care and experiments was approved by the Animal Care and Use Committee of Southern Medical University (protocol #SMUL2022189). All animal studies were complied with relevant ethical regulations for animal testing and research.

Note that full information on the approval of the study protocol must also be provided in the manuscript.

Flow Cytometry

Plots

Confirm that:

- [x] The axis labels state the marker and fluorochrome used (e.g. CD4-FITC).
- [x] The axis scales are clearly visible. Include numbers along axes only for bottom left plot of group (a 'group' is an analysis of identical markers).
- [x] All plots are contour plots with outliers or pseudocolor plots.
- [x] A numerical value for number of cells or percentage (with statistics) is provided.

Methodology

Sample preparation	Tumors were dissected from mice, cut into small pieces and digested in RPMI 1640 supplemented with 10% FBS, DNase I (0.1mg/ml) and Collagenase IV (0.5mg/ml) at 37℃ for 30 minutes with gentle shaking. The mixtures were filtered through 75-µm cell strainers and separated by centrifugation (300g x 5 minutes) to harvest the single cells. Thereafter, the cells were resuspended in PBS supplemented with 2% FBS and used for subsequent experiments.

Instrument	All FACS analyses were perfomed on the BD LSRFortessa (BD Biosciences)
Software	FlowJo software (version 10.5; Tree Star)
Cell population abundance	The frequencies of the key cell populations and the gates used to identify specific cell populations are shown in the source data.
Gating strategy	For all FACS experiments, the main population was gated using FSC-A and SSC-A. Fluorophores were measured using their correct lasers and gating was determined based on control samples for all experiments.

- [x] Tick this box to confirm that a figure exemplifying the gating strategy is provided in the Supplementary Information.
